# Tetraploidy‐linked sensitization to CENP‐E inhibition in human cells

**DOI:** 10.1002/1878-0261.13379

**Published:** 2023-02-11

**Authors:** Koya Yoshizawa, Akira Matsura, Masaya Shimada, Sumire Ishida‐Ishihara, Fuyu Sato, Takahiro Yamamoto, Kan Yaguchi, Eiji Kawamoto, Taruho Kuroda, Kazuya Matsuo, Nobuyuki Tamaoki, Ryuichi Sakai, Yasuhito Shimada, Mithilesh Mishra, Ryota Uehara

**Affiliations:** ^1^ Graduate School of Life Science Hokkaido University Sapporo Japan; ^2^ Faculty of Advanced Life Science Hokkaido University Sapporo Japan; ^3^ Graduate School of Medicine Mie University Tsu Japan; ^4^ Faculty of Molecular Chemistry and Engineering Kyoto Institute of Technology Kyoto Japan; ^5^ Research Institute for Electronic Science Hokkaido University Sapporo Japan; ^6^ Graduate School and Faculty of Fisheries Sciences Hokkaido University Sapporo Japan; ^7^ Department of Integrative Pharmacology Mie University Graduate School of Medicine Tsu Japan; ^8^ Department of Biological Sciences Tata Institute of Fundamental Research Mumbai India

**Keywords:** chromosome, mitosis, motor protein, ploidy

## Abstract

Tetraploidy is a hallmark of cancer cells, and tetraploidy‐selective cell growth suppression is a potential strategy for targeted cancer therapy. However, how tetraploid cells differ from normal diploids in their sensitivity to anti‐proliferative treatments remains largely unknown. In this study, we found that tetraploid cells are significantly more susceptible to inhibitors of a mitotic kinesin (CENP‐E) than are diploids. Treatment with a CENP‐E inhibitor preferentially diminished the tetraploid cell population in a diploid–tetraploid co‐culture at optimum conditions. Live imaging revealed that a tetraploidy‐linked increase in unsolvable chromosome misalignment caused substantially longer mitotic delay in tetraploids than in diploids upon moderate CENP‐E inhibition. This time gap of mitotic arrest resulted in cohesion fatigue and subsequent cell death, specifically in tetraploids, leading to tetraploidy‐selective cell growth suppression. In contrast, the microtubule‐stabilizing compound paclitaxel caused tetraploidy‐selective suppression through the aggravation of spindle multipolarization. We also found that treatment with a CENP‐E inhibitor had superior generality to paclitaxel in its tetraploidy selectivity across a broader spectrum of cell lines. Our results highlight the unique properties of CENP‐E inhibitors in tetraploidy‐selective suppression and their potential use in the development of tetraploidy‐targeting interventions in cancer.

## Graphical abstract

Tetraploidy arising through whole‐genome duplication is a cancer hallmark. Inhibitors of a mitotic kinesin CENP‐E preferentially suppress tetraploid cells over diploid counterparts with superior generality across different cellular backgrounds. Comparative imaging reveals tetraploidy‐linked aggravation of chromosome misalignment, mitotic delay/catastrophe and subsequent cell death upon CENP‐E inhibition, demonstrating the ‘Achilles' heel' of cell division control with drastically increased chromosome number.

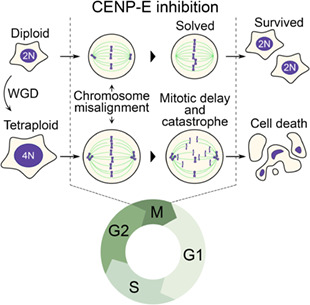

**Abbreviations**
CENP‐E: centromere‐associated protein E
DAPI: 4′,6‐diamidino‐2‐phenylindole
DMEM: Dulbecco's modified Eagle's medium
IC_50_
: 50% inhibition concentration
IMDM: Iscove's modified Dulbecco's medium
NEBD: nuclear envelope breakdown
PCEI‐HU: a photo‐switchable CENP‐E inhibitor
PSS: photostationary state
SAC: spindle assembly checkpoint
STLC: S‐trityl‐l‐cysteine
TTBS: Tween Tris‐buffered saline
WGD: whole‐genome duplication

## 1 Introduction

Tetraploidy resulting from whole‐genome duplication (WGD) of a normal diploid cell is a common hallmark of cancer. Recent cancer genome analyses revealed that about 30% of solid tumors had undergone at least one round of WGD [1, 2]. The induction of tetraploidization facilitates tumorigenesis and malignant transformation in mice models, suggesting that tetraploidy is a critical intermediate state in these pathogenic processes [3, 4]. The principle of tetraploidy‐driven cancer formation is still largely unknown. However, recent studies have proposed that increased tolerance to chromosome alterations and instability or enhanced invasiveness upon tetraploidization contribute to the oncogenic quality of tetraploid cells [5, 6, 7]. Because of the commonality and significant contributions of tetraploidy to the tumorigenic process, selective suppression of tetraploid cell growth is a promising strategy for cancer chemotherapy [8]. In this context, mitosis is a good candidate for tetraploidy‐selective chemotherapeutic targets. A previous study reported that tetraploid hTERT‐RPE1 cells took longer to go through mitosis than diploid counterparts even when they had the normal number (i.e. 2) of centrosomes [9], suggesting that the doubled number of chromosomes increases the burden on the mitotic mechanism upon tetraploidization.

Moreover, recent studies have revealed that tetraploid cell lines are more susceptible to anti‐mitotic microtubule stabilizer paclitaxel or inhibitors of mitotic kinase MPS1, Plk1 or mitotic kinesin motor protein Kif18A when compared with their diploid counterparts [10, 11, 12, 13]. These findings suggest that tetraploid cells have an increased dependence on specific aspects of mitotic regulations, presumably as adaptive mechanisms to the increased burden of doubled chromosomes. On the other hand, other studies have reported tetraploidy‐ or polyploidy‐associated increases in resistance to cytotoxic drugs, including anti‐mitotic compounds such as paclitaxel or doxorubicin [14, 15]. This discrepancy between different reports may reflect heterogeneous drug responses of tetraploid cells with different backgrounds, potentially limiting the generality of tetraploidy‐selective efficacy of anti‐mitotic compounds. Further elucidation of the principles of tetraploidy‐linked changes in drug responses would provide more choices of tetraploidy‐selective cell growth suppression, potentially overcoming the limited generality of the tetraploidy‐selective efficacy and benefiting the development of tetraploidy‐targeting chemotherapeutic strategy in broad cancer types.

Centromere‐associated protein E (CENP‐E; kinesin‐7) is a mitotic kinesin that plays an essential role in transporting mitotic chromosomes along spindle microtubules and aligning them on the equatorial metaphase plate [16, 17, 18]. Inhibition of CENP‐E ATPase activity by an allosteric inhibitor GSK‐923295 causes tight binding of the protein to microtubules, resulting in frequent chromosome misalignment at the spindle poles and mitotic arrest through activation of the spindle assembly checkpoint (SAC) [18, 19, 20]. The specific requirement of CENP‐E in mitosis makes it an ideal candidate for an anti‐mitotic cancer therapeutic target [21, 22]. In mitosis, not all chromosomes require CENP‐E activity for their alignment. Whereas the large population of mitotic chromosomes can align at the equatorial plate, those initially located in the nuclear peripheral region upon mitotic entry tend to be trapped at the spindle pole in the absence of CENP‐E activity [23]. Moreover, whereas smaller‐sized chromosomes tend to re‐align to the equatorial plate even when initially trapped at the spindle poles, larger‐sized chromosomes have less chance of re‐alignment [24]. Therefore, the location and size of the mitotic chromosomes affect their susceptibility to CENP‐E inhibition. However, it remains unclear whether and how drastic differences in chromosome number affect cellular susceptibility to CENP‐E inhibition.

In this study, we compared the effect of anti‐mitotic compounds on the proliferation of cells at different ploidy states. Among these compounds, CENP‐E inhibitors significantly suppressed the proliferation of tetraploid cells compared with diploids in different culture conditions or cellular backgrounds. We found that the tetraploidy‐selective suppression was based on the aggravation of chromosome misalignment, mitotic arrest and consequent cell death upon CENP‐E inhibition. On the other hand, paclitaxel caused tetraploidy‐selective cell death via the aggravation of mitotic spindle multipolarization, highlighting the difference in the principle of tetraploidy‐selective cell growth suppression by paclitaxel and CENP‐E inhibitors. We also found that a CENP‐E inhibitor showed selectivity toward a broader spectrum of tetraploid cell lines compared with paclitaxel, demonstrating superior generality of CENP‐E‐targeted tetraploidy suppression. Based on our results, we discuss the potential values of various tetraploidy‐targeting mechanisms of different anti‐mitotic compounds.

## 2 Materials and methods

### 2.1 Cell culture and flow cytometry

HAP1 cells (RRID: CVCL_Y019; from Haplogen GmbH, Vienna, Austria) [25] were cultured in Iscove's modified Dulbecco's medium (IMDM; Wako Pure Chemical Industries, Osaka, Japan) or Dulbecco's modified Eagle's Medium (DMEM; Wako). HCT116 cells (WT line, RRID: CVCL_0291 from Riken BRC, Ibaraki, Japan, and p53 knock‐out line, CVCL_HD97 from Horizon Discovery, Cambridge, UK) were cultured in McCoy's 5A (Wako) or DMEM. The hTERT‐RPE1 cells (RRID: CVCL_4388; a kind gift from Dr Gohta Goshima) were cultured in DMEM/Ham's F‐12 (Wako). RKO cells (RRID: CVCL_0504; a kind gift from Dr David Pellman) were cultured in McCoy's 5A. DLD1 cells (RRID: CVCL_A3YG; from Riken BRC) were cultured in DMEM with sodium pyruvate (Wako). Media for all cell lines were supplemented with 10% FBS and 1× antibiotic‐antimycotic solution (AA; Sigma‐Aldrich, St. Louis, MO, USA). All cells were periodically authenticated by morphologic inspection in the past 3 years. All experiments were performed with mycoplasma‐free cells, tested by Hoechst 33342 (Dojindo, Kumamoto, Japan) or 4′,6‐diamidino‐2‐phenylindole (DAPI; Dojindo) staining.

Haploid HAP1 cells were maintained by size‐based cell sorting, and diploid or tetraploid HAP1 lines and tetraploid hTERT‐RPE1 cells were established as previously described [26]. To establish tetraploid HCT116, RKO or DLD1 cell lines, parental diploids were treated with 40 ng·mL^−1^ nocodazole for 4 h, washed three times with cell culture medium, shaken off and treated with 10 μg·mL^−1^ cytochalasin B for 4 h. Cells were then washed three times with cell culture medium and diluted in 10‐cm dishes. After 7–9 days, colonies containing cells that were uniform in size and larger than diploids were clonally expanded and checked for DNA content to select near‐tetraploid clones. For induction of acute polyploidization in HAP1 cells, diploid cells were treated with 400 nm VX‐680 for 16 h, washed three times with cell culture medium, further incubated for 30 h to restore cell proliferation, and subjected to the co‐culture experiment (see below). The DNA content of the VX‐680‐treated cells was tested immediately after the 16‐h drug treatment by flow cytometry. For DNA content analyses, 2 × 10^6^ cells were stained with 10 μg·mL^−1^ Hoechst 33342 for 15 min at 37 °C, and DNA content was analyzed using a JSAN desktop cell sorter (Bay Bioscience, Brookline, MA, USA).

### 2.2 Inhibitors

Inhibitors were purchased from the following distributors: AdooQ BioScience (Irvine, CA, USA; Aurora A inhibitor I, BI‐2536, epothilone A, MK‐1775 and VX‐680); Axon Medchem (Reston, VA, USA; SPL‐B); Focus Biomolecules (Plymouth Meeting, PA, USA; Latrunculin A); MedChemExpress (Princeton, NJ, USA; PF‐2771); Selleck Chemicals (Houston, TX, USA; GSK‐923295); Sigma‐Aldrich (Importazole, RO‐3306, and S‐trityl‐l‐cysteine); Thermo Fisher Scientific (Waltham, MA, USA; Colcemid (KaryoMAX Colcemid)); Calbiochem (San Diego, California CA, USA; Etoposide); LKT Laboratories (St. Paul, MN, USA; Vinblastine); Tocris Bioscience, (Bristol, UK; Monastrol); Wako (Cytochalasin B, daunorubicin, doxorubicin, nocodazole and paclitaxel).

### 2.3 Colorimetric cell proliferation assay

For cell viability assay, cells were seeded on 96‐well plates at the densities described below: haploid, diploid or tetraploid HAP1 cells at 2250, 1125 or 562.5 cells per well, respectively; diploid or tetraploid HCT116 cells (WT or p53 knock‐out) at 1350 or 675 cells per well, respectively; diploid or tetraploid hTERT‐RPE1 cells at 360 or 180 cells per well, respectively; diploid or tetraploid RKO cells at 1350 or 675 cells per well, respectively; and diploid or tetraploid DLD1 cells at 900 or 450 cells per well, respectively.

After 24 h, cells were treated with different concentrations of anti‐mitotic compounds. Either 44 (HAP1 cells) or 68 h (HCT116 cells, hTERT‐RPE1 cells, RKO cells, or DLD1 cells) after the addition of the compounds, 5% Cell Counting Kit‐8 (Dojindo) was added to the culture, incubated for 4 h, and absorbance at 450 nm was measured using the Sunrise plate reader (Tecan, Männedorf, Switzerland). A 50% inhibition concentration (IC_50_) was calculated by curve fitting of normalized dose–response data using nonlinear regression:

$$y=d+\frac{a-d}{1+{\left(\frac{x}{c}\right)}^{b}}$$

where *y* is the normalized absorbance, *x* is drug concentration, *a* or *d* is the absorbance at zero or infinite drug concentration, respectively, and b or c is the slope factor or the inflection point, respectively. In all cell proliferation assays, we tested two replicates in each independent experimental trial and analyzed eight data from four independent experiments for each condition. All datasets obtained in cell proliferation assays and all coefficients and constant values obtained by the curve fitting are presented in Table S1.

### 2.4 Mixed culture experiment

For flow cytometry analysis, EGFP‐labeled diploid and non‐labeled tetraploid or acutely formed polyploid HAP1 cell suspension (1.5 × 10^4^ cells·mL^−1^ each) were mixed in a 1 : 1 ratio, with 1.8 mL seeded on 6‐well plates coated with collagen type I (Corning, Corning, NY, USA). After 24 h, paclitaxel or GSK‐923295 was treated in the co‐culture. At 48 h after the addition of the compounds, cells were trypsinized, suspended in DPBS, stained with 10 μg·mL^−1^ Hoechst 33342, and analyzed by flow cytometry. The two mixed cell populations were counted separately based on the EGFP fluorescence signal. For prolonged analyses of the co‐culture, cells were passaged every 2 days, at which times paclitaxel or GSK‐923295 was newly introduced into the culture at a constant concentration.

For live imaging, diploid and tetraploid cells stably expressing histone H2B transgene tagged with EGFP and mCherry, respectively, were mixed in a 1 : 1 ratio (1.35 × 10^4^ cells·mL^−1^ each), 0.2 mL seeded on collagen‐coated 8‐well imaging chamber. After 24 h, paclitaxel or GSK‐923295 was treated in the co‐culture, and live imaging was subsequently conducted for 48 h. The first mitotic events after the drug treatment were analyzed.

### 2.5 Immunofluorescence staining

Cells were fixed with 100% methanol at −20 °C for 10 min, treated with BSA blocking buffer (150 mm NaCl, 10 mm Tris–HCl pH 7.5, 5% BSA and 0.1% Tween 20) for 30 min at 25 °C, incubated with rat monoclonal anti‐α‐tubulin (YOL1/34, EMD Millipore, Burlington, MA, USA; 1 : 1000), mouse monoclonal anti‐PCNT (ab28144, Abcam, Cambridge, UK; 1 : 1000), rabbit polyclonal anti‐CP110 (A301‐343A, Bethyl Laboratories, Montgomery, TX, USA; 1 : 1000) overnight at 4 °C, and with fluorescence (Alexa Fluor 488, 568, 647)‐conjugated secondaries (Jackson ImmunoResearch Laboratories, West Grove, PA, USA or Abcam; 1 : 1000) overnight at 4 °C at indicated dilutions. DNA was stained with 0.5 μg·mL^−1^ DAPI. Following each treatment, cells were washed three times with phosphate‐buffered saline.

### 2.6 Microscopy

Fixed cells were observed under a TE2000 microscope (Nikon, Tokyo, Japan) equipped with a ×100 1.4 NA Plan‐Apochromatic, a CSU‐X1 confocal unit (Yokogawa, Tokyo, Japan) and an iXon3 electron multiplier‐charge coupled device (EMCCD) camera (Andor, Belfast, UK) or ORCA‐ER CCD camera (Hamamatsu Photonics, Tokyo, Japan), or with a Ti2 microscope (Nikon) with ×60 1.4 NA Apochromatic, and Zyla4.2 sCMOS camera (Andor). Live cell imaging was conducted at 37 °C with 5% CO_2_ using a Ti‐2 microscope with ×20 0.75 NA Plan‐Apochromatic and Zyla4.2. For live imaging, cells were cultured in phenol red‐free IMDM (Thermo Fisher Scientific) supplemented with 10% FBS and 1× AA. Image acquisition was controlled by μManager (an open‐source software program)[27].

### 2.7 Photo‐switching CENP‐E inhibition experiment

A 1‐mm stock solution of PCEI‐HU, a photo‐switchable CENP‐E inhibitor, in DMSO was diluted at 1 : 2000 in IMDM in a microtube, then irradiated with 365 nm LED light (Asahi Spectra, Tokyo, Japan, 416 mW·cm^−2^ at 100%, irradiated from 5 cm above the sample for 60 s) to reach a photostationary state (PSS) enriched in non‐inhibitory *cis* isomer, and then immediately treated in diploid or tetraploid HAP1 cells at the final concentration of 0.5 μm. At the same time, cells were co‐treated with 10 μm MG132 (Peptide Institute; for blocking anaphase onset) and 100 nm SiR‐DNA (Cytoskeleton Inc., Denver, CO, USA; for visualizing mitotic chromosomes). After a 2‐h incubation in the dark, we started far‐red fluorescence live imaging of SiR‐DNA‐stained mitotic chromosomes. Note that observing light for live imaging does not affect the photoisomerization of PCEI‐HU [28]. At 15 min after the initiation of live imaging, PCEI‐HU‐treated cells were irradiated with a 505‐nm LED light (Asahi Spectra, 141 mW·cm^−2^ at 100%, irradiated from 3.2 cm above the sample for 35 s) to make the compound reach a PSS enriched in inhibitory *trans* isomer of PCEI‐HU. We then traced the motion of mitotic chromosomes pre‐misaligned from or pre‐aligned at the metaphase plate at the time of 505‐nm light irradiation.

### 2.8 Immunoblotting

Cells were lysed in SDS/PAGE sample buffer (1.125% SDS, 35 mm Tris–HCl, pH 6.8, 11.25% glycerol, 5% 2‐mercaptoethanol), boiled for 5 min, and subjected to SDS/PAGE. Separated proteins were transferred on to Immun‐Blot PVDF membrane (Bio‐Rad, Hercules, CA, USA). The blotted membranes were blocked with 0.3% skim milk in Tween Tris‐buffered saline (TTBS; 50 mm Tris, 138 mm NaCl, 2.7 mm KCl, and 0.1% Tween 20), incubated with mouse monoclonal anti‐β‐tubulin (10G10, Wako; 1 : 5000) or mouse monoclonal anti‐p53 (K0181‐3, MBL, Tokyo, Japan; 1 : 1000) antibodies overnight at 4 °C, and incubated with horseradish peroxidase‐conjugated anti‐mouse secondary antibodies (115‐035‐003, Jackson ImmunoResearch Laboratories; 1 : 2000) for 1 h at 37 °C. Each step was followed by three washes with TTBS. For signal detection, the ezWestLumi plus ECL Substrate (ATTO, Tokyo, Japan) and a LuminoGraph II chemiluminescent imaging system (ATTO) were used.

### 2.9 Statistical analysis

All quantitative data subjected to statistical analyses in this study were abnormally distributed in the Shapiro–Wilk test. To compare two data groups not assumed to have equal variances, we used the Brunner–Munzel test. To compare more than two groups of data with equal or unequal sample sizes, we used the Steel–Dwass test or the Dwass–Steel–Critchlow–Fligner (DSCF) test, respectively. When comparing a common diploid control with each of multiple tetraploid samples, we used the Steel test. Multiple group analyses of drug IC_50_ differences among haploid, diploid and tetraploid cells were conducted using the Kruskal–Wallis test with a *post‐hoc* Steel–Dwass test. Statistical significance was set at *P* < 0.05 for all analyses. The compounds with the effect size of Kruskal–Wallis test $\epsilon^{2}>0.655$ were defined as ‘significantly ploidy‐selective’ [29]. All statistical analyses were conducted with r software (4.2.1; The R Foundation, Vienna, Austria) using brunnermunzel, minpack.lm, PMCMRplus, rcompanion, Rmisc, nparcomp, rstatix and stats packages.

## 3 Results

### 3.1 Selective suppression of tetraploid cell growth by CENP‐E inhibitors

**Fig. 1:**
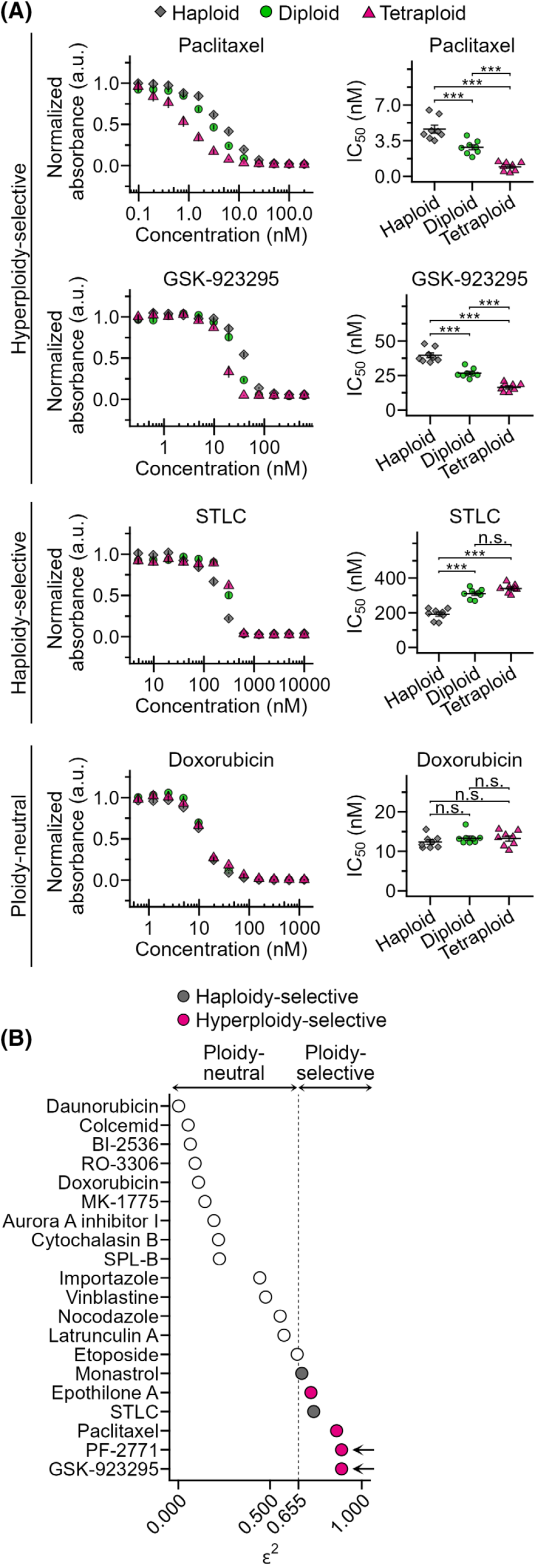
Identification of ploidy‐selective anti‐mitotic compounds. (A) Dose–response curve of normalized absorbance (left) and calculated IC_50_ values (right) in a comparative colorimetric cell proliferation assay using anti‐mitotic compounds in haploid, diploid and tetraploid HAP1 cells. Mean ± standard error (SE) of eight replicates from four independent experiments for each condition. Asterisks indicate statistically significant differences in IC_50_ between cells with different ploidies (***P < 0.001, n.s.: not significant, the Steel–Dwass test). See also Fig. S2 and Table S1 for data of all compounds tested. (B) Evaluation of ploidy selectivity of different anti‐mitotic compounds based on effect size *ε* ^*2*^ of ploidy‐linked IC_50_ differences obtained by analyzing data in Fig. S2 using the Kruskal–Wallis test (eight replicates from four independent experiments were analyzed). Inhibitors that have significant ploidy‐dependent differences in their efficacy (*ε* ^2^ > 0.655) with positive linear correlations are categorized as ‘hyperploidy‐selective’, and those with significantly higher efficacy toward haploids as ‘haploidy‐selective’. Arrows indicate CENP‐E inhibitors.

To understand the influence of ploidy difference on cellular sensitivity to mitotic perturbations, we compared the effect of various anti‐mitotic compounds on isogenic haploid, diploid, and tetraploid HAP1 cells [26] (Fig. S1A) using a colorimetric cell proliferation assay. Different compounds showed diverse trends and varying degrees of ploidy dependency in efficacy (Figs 1A and S2A,B). Therefore, we categorized these compounds based on statistical significance and type of ploidy‐linked differences in their IC_50_ values (Figs 1A,B and S2B; see also Section 2). Among the compounds that showed significant ploidy‐linked changes in efficacy, a microtubule‐stabilizing compound, paclitaxel, had higher efficacy against cells with higher ploidy (hyperploidy‐selective; Figs 1A,B and S2B), consistent with the previous study [11]. CENP‐E inhibitors GSK‐923295 and PF‐2771 were also remarkably hyperploidy‐selective (Figs 1A,B and S2B). Hyperploidy‐selective suppression by CENP‐E inhibitors was also observed in another tetraploid HAP1 cell line (Fig. S3A,B). In contrast, Eg5 inhibitors S‐trityl‐l‐cysteine (STLC) and monastrol suppressed haploid cells more efficiently than diploids, although its efficacy was equivalent between diploids and tetraploids (haploidy‐selective; Figs 1A,B and S2B). The topoisomerase II inhibitors daunorubicin and doxorubicin tended to suppress the proliferation of cells with different ploidies with equivalent efficacy (Figs 1A and S2B). Diverse profiles of ploidy‐linked changes in the efficacy of different anti‐mitotic compounds indicate that ploidy difference has complex and non‐uniform effects on different aspects of molecular regulations of cell division. The ploidy‐linked change in the efficacy of CENP‐E inhibitors was particularly notable and has not previously been reported. Therefore, we decided to address further the significance and mechanism of tetraploidy selectivity of CENP‐E inhibitors in comparison with paclitaxel, a previously reported tetraploidy‐selective compound [11].

**Fig. 2:**
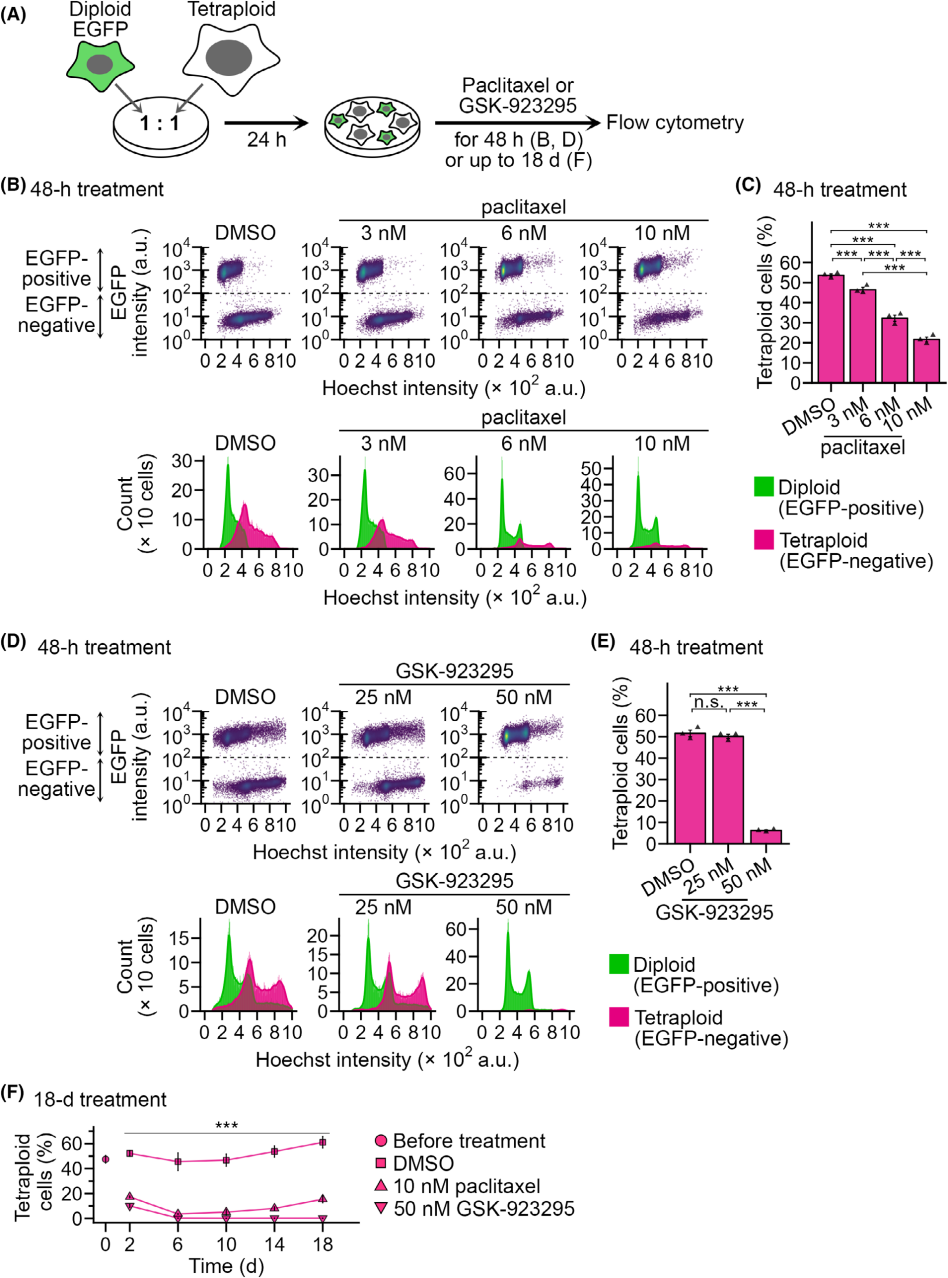
Selective suppression of tetraploid HAP1 cells in diploid‐tetraploid co‐culture by paclitaxel or GSK‐923295. (A) Scheme of diploid‐tetraploid co‐culture experiment. (B,D) Flow cytometric analyses of diploid and tetraploid cell numbers in their co‐culture treated with different concentrations of paclitaxel (B) or GSK‐923295 (D) for 48 h. Dot plots of EGFP intensity against the Hoechst signal (corresponding to DNA content) and histograms of the Hoechst signal are shown at top and bottom, respectively. Cell populations originating from diploid or tetraploid cells were distinguished based on EGFP signal intensity and are displayed separately in the histograms. Representative data from three independent experiments. (C,E) The proportion of tetraploid cells in the diploid‐tetraploid co‐culture. Mean ± SE of three independent experiments for each condition. Asterisks indicate statistically significant differences between conditions (***P < 0.001, the Steel–Dwass test). (F) Time course of tetraploid proportion in diploid‐tetraploid co‐culture treated with 10 nm paclitaxel or 50 nm GSK‐923295. The data point at day 0 corresponds to the initial tetraploid proportion before adding the compounds. Mean ± SE of three independent experiments for each condition. Asterisks indicate statistically significant differences from DMSO‐treated control (***P < 0.001, the Steel test). See also Fig. S4C for the corresponding flow cytometry data.

We next investigated the effect of paclitaxel and GSK‐923295 on cell proliferation in 1 : 1 co‐culture of EGFP‐labeled diploid and unlabeled tetraploid HAP1 cells (Figs 2A and S1A). Flow cytometric analysis revealed that DMSO‐treated co‐culture roughly kept the original diploid‐tetraploid ratio after 48 h of treatment (Fig. 2B–E), demonstrating that diploid and tetraploid cells proliferated at a similar rate in this condition. On the other hand, 3–10 nm paclitaxel significantly reduced tetraploid proportion in the co‐culture in a dose‐dependent manner (Figs 2B,C and S4A,B). GSK‐923295 also caused a drastic reduction of tetraploid proportion in the co‐culture when treated at 50 nm (Fig. 2D,E). Recent studies have reported that genome instability induced by mitotic errors drove chemoresistance to various cytotoxic compounds, including paclitaxel, when cells were continuously treated with these compounds for a few weeks [30, 31]. Therefore, we tested whether paclitaxel and GSK‐923295 maintained the trend of tetraploidy selectivity for a longer period (up to 18 days) in the diploid and tetraploid HAP1 co‐culture (Figs 2F and S4C). In the prolonged paclitaxel‐treated co‐culture, the tetraploid population continuously decreased for the first 6 days, after which we observed a weak but reproducible trend of reversion of the tetraploid ratio in the co‐culture (Figs 2F and S4C). In contrast, 50 nm GSK‐923295 treatment continuously decreased the tetraploid population without reversion, almost entirely decimating it in 18 days (Figs 2F and S4C). These data illustrate the high potential of CENP‐E as a target for tetraploidy‐selective suppression within heterogeneous cell populations.

**Fig. 3:**
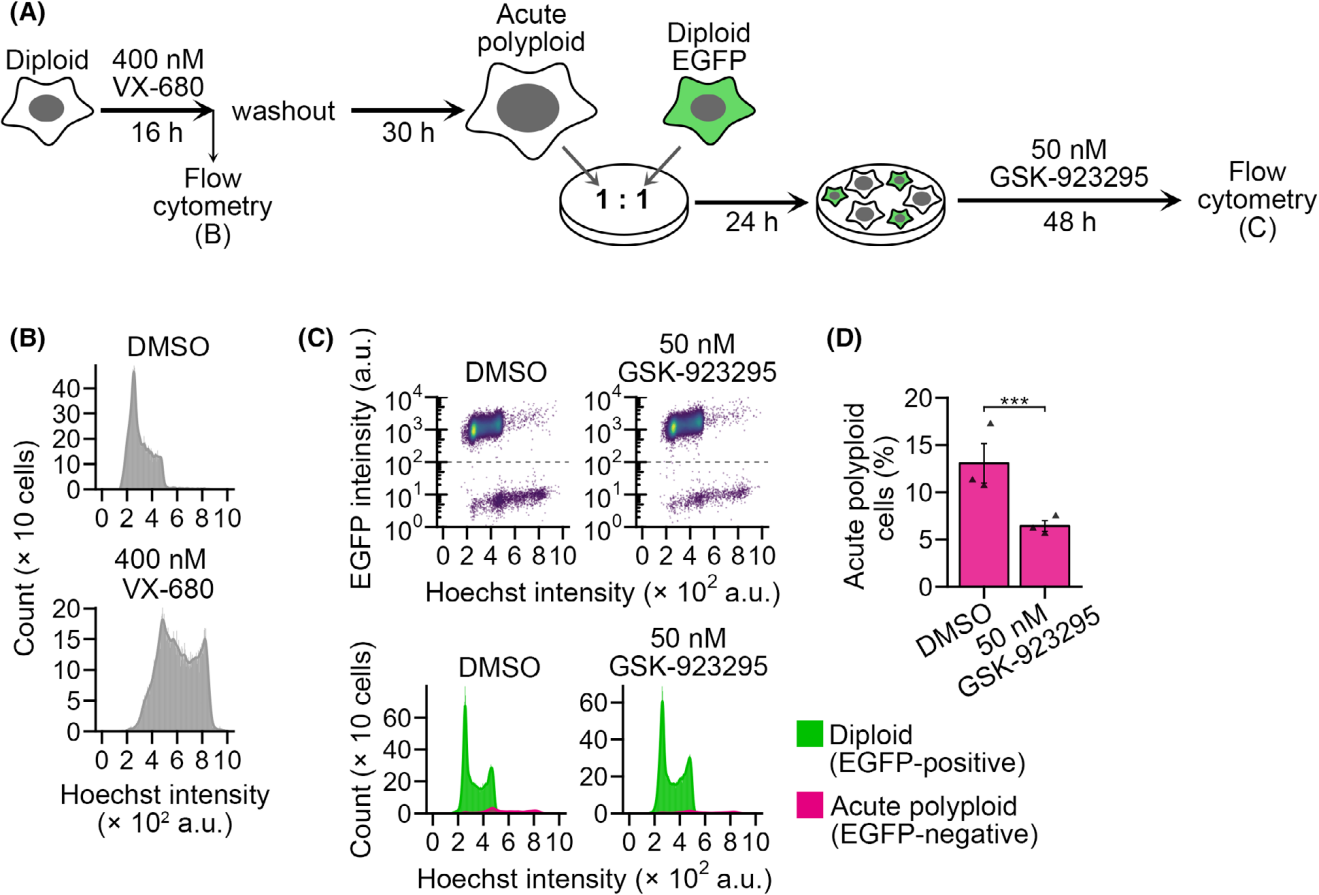
Selective suppression of acute polyploid HAP1 cells in co‐culture by GSK‐923295. (A) Scheme of diploid‐acute polyploid co‐culture experiment. (B) Flow cytometric analyses of DNA content in HAP1 cells immediately after 16 h of VX‐680 treatment. Representative data from three independent experiments. (C) Flow cytometric analyses of diploid and acute polyploid cell numbers in their co‐culture treated with 50 nm GSK‐923295 for 48 h. Dot plots of EGFP intensity against the Hoechst signal (corresponding to DNA content) and histograms of the Hoechst signal are shown at top and bottom, respectively. Cell populations originating from diploid or acute polyploid cells were distinguished based on EGFP signal intensity and are displayed separately in the histograms. Representative data from three independent experiments. (D) The proportion of acute polyploid cells (EGFP‐negative) in the co‐culture. Mean ± SE of three independent experiments for each condition. Asterisks indicate statistically significant differences between conditions (***P < 0.001, the Brunner–Munzel test).

We further addressed the generality of the hyperploidy‐selectivity of CENP‐E inhibition by investigating the effect of GSK‐923295 on the proliferation of acutely induced polyploid cell populations. We induced acute polyploidization of diploid HAP1 cells by treating a pan‐aurora kinase inhibitor VX‐680 and co‐cultured them with non‐treated EGFP‐labeled diploid cells at a 1 : 1 ratio in the absence or presence of GSK‐923295 (Fig. 3A,B; see Section 2 for details). In the control co‐culture, the proportion of the polyploid population became 13% after 48 h of incubation, reflecting the slower proliferation of the acutely polyploidized cells than diploids (Fig. 3C,D). In GSK‐923295‐treated co‐culture, the polyploid proportion significantly decreased (to 6%) compared with control (Fig. 3C,D). CENP‐E inhibition thus can select acutely formed polyploid cells as well as chronically established tetraploid cells.

### 3.2 Tetraploidy‐linked aggravation of chromosome misalignment, mitotic arrest and subsequent cohesion fatigue upon CENP‐E inhibition

**Fig. 4:**
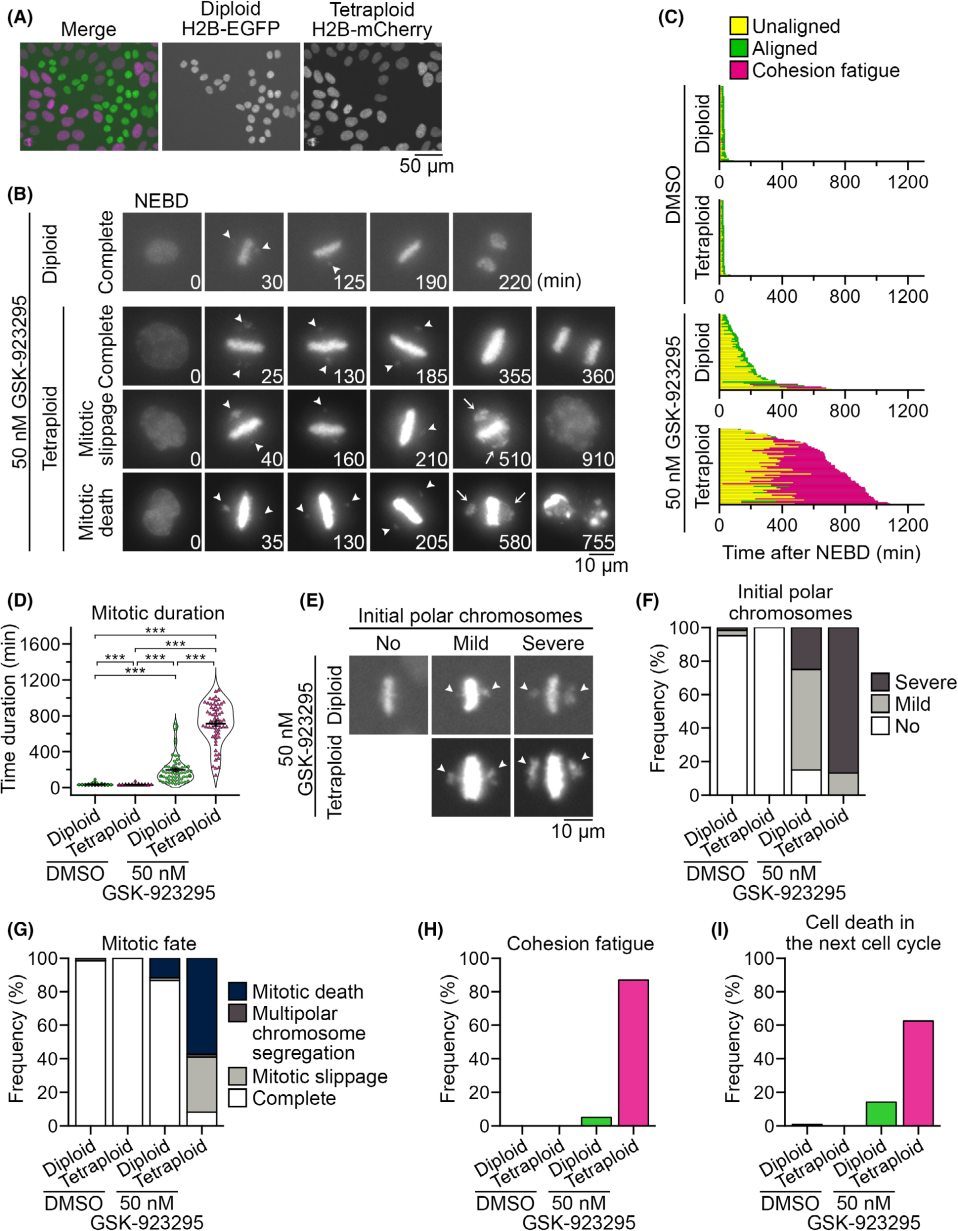
Tetraploidy‐linked aggravation of chromosome misalignment and mitotic failure upon GSK‐923295 treatment. (A) Fluorescence microscopy of co‐cultured diploid and tetraploid HAP1 cells expressing histone H2B‐EGFP and histone H2B‐mCherry, respectively. Representative data from two independent experiments. (B) Time‐lapse images of the mitotic progression of GSK‐923295‐treated diploid or tetraploid cells in the co‐culture. Arrowheads: misaligned polar chromosomes. Arrows: Gross chromosome scattering caused through cohesion fatigue. Representative data from two independent experiments. (C) Analysis of mitotic progression of control and GSK‐923295‐treated diploid or tetraploid cells in (B). Each bar represents a single mitotic event (from NEBD to anaphase onset or mitotic exit) in a dividing cell. At least 60 cells from two independent experiments were analyzed for each condition. (D) Mitotic duration (from NEBD to anaphase onset or mitotic exit) in control and GSK‐923295‐treated diploid or tetraploid cells in (B). Mean ± SE of at least 60 cells from two independent experiments for each condition. Asterisks indicate statistically significant differences between conditions (***P < 0.001, the DSCF test). (E) Different degrees of polar chromosome misalignment appeared upon the formation of the metaphase plates (initial polar chromosomes; arrowheads) in GSK‐923295‐treated diploid or tetraploid cells. Representative data from two independent experiments. (F–I) Frequency of different degrees of initial polar chromosome misalignment (F), mitotic fates (G), cohesion fatigue event (H) and cell death in the subsequent cell cycle (I) in control and GSK‐923295‐treated diploid or tetraploid cells in (B). At least 60 cells (F–H) and 32 cells (I) from two independent experiments were analyzed for each condition.

To understand the cause of the tetraploidy‐selective growth suppression by CENP‐E inhibition, we conducted live imaging of the mitotic progression in co‐cultured diploid and tetraploid HAP1 cells. Diploid and tetraploid cells were differentially labeled by stably expressing histone H2B transgene tagged with EGFP and mCherry, respectively (Figs 4A,B and S1A). In DMSO‐treated co‐culture, diploid and tetraploid cells underwent normal cell division with an average mitotic duration of 34 and 30 min (from nuclear envelope breakdown (NEBD) to anaphase onset), respectively (Fig. 4C,D; Videos S1 and S2), which was consistent with a similar mitotic stage distribution between asynchronous diploids and tetraploids in fixed cell images (Fig. S5A). When treated with 50 nm GSK‐923295, which caused sharp tetraploidy‐selective suppression (Fig. 2E), diploid and tetraploid cells manifested a characteristic mitotic defect typically observed in CENP‐E inhibited cells: a subset of mitotic chromatids was captured at the spindle poles, while the remaining majority completed congression to the spindle midzone (defined as ‘polar chromosomes’ phenotype in Fig. 4B,E,F) [20, 32]. We observed this defect at a high frequency in the early mitotic stage both in diploids and tetraploids treated with GSK‐923295 (85% and 100%, respectively; Fig. 4E,F; Videos S3 and S4). In most GSK‐923295‐treated diploid cells, these polar chromosomes gradually moved into the metaphase plate, and all chromosomes eventually aligned (Fig. 4B,C). As a result, the majority of diploid cells (87%) entered anaphase and completed cell division despite considerable mitotic delay (with an average mitotic duration of 197 min; Fig. 4D,G; Video S3). Compared with diploids, GSK‐923295‐treated tetraploid cells manifested more severe polar chromosome misalignment (Fig. 4E,F). In most cases, these polar chromosomes also gradually moved into the metaphase plate but never completed the alignment (Figs 4B and S5B–D; Video S4). As a result, GSK‐923295‐treated tetraploid cells spent an extremely long time in mitosis (with an average mitotic duration of 713 min) and 87% of them eventually underwent cohesion fatigue (catastrophic chromosome scattering) [33] (Fig. 4B,C,H). Cohesion fatigue took place 347 ± 15 min after NEBD (mean ± standard error, *n* = 53 from two independent experiments) in GSK‐923295‐treated tetraploid cells when most GSK‐923295‐treated diploids had completed congression of initially misaligned chromosomes and entered anaphase (Fig. 4C). Subsequent to cohesion fatigue, GSK‐923295‐treated tetraploid cells either died during mitosis or exit mitosis without chromosome segregation (mitotic slippage; Fig. 4B,G). A substantial proportion of GSK‐923295‐treated tetraploid cells (63%) that exited mitosis died during the next cell cycle (Fig. 4I). In contrast, most GSK‐923295‐treated diploids survived the next cell cycle despite the delay in the previous mitosis. These results suggest that ploidy‐dependent difference in time duration of mitotic arrest critically affects the fate of CENP‐E‐inhibited cells: while diploid cells resolve mitotic arrest within the critical time window for chromatid cohesion maintenance in the above CENP‐E inhibitory condition, tetraploids go beyond that time window and suffer catastrophic mitotic damages.

A recent study revealed that tetraploid cells were particularly defective in retention of pre‐aligned metaphase chromosomes upon inhibition of a mitotic kinesin Kif18A, highlighting the unstable nature of the metaphase plate in tetraploid cells [12]. This prompted us to test the effect of CENP‐E inhibition on the retention of pre‐aligned chromosomes in diploid and tetraploid cells. For this, we used a previously developed photo‐switchable CENP‐E inhibitor (PCEI‐HU), which reversibly converts to non‐inhibitory *cis* or inhibitory *trans* isomer by irradiating UV or visible light, respectively [28] (Fig. S6A). Diploid and tetraploid cells were treated with the inhibitor at the photo‐stationary state (PSS) enriched in the non‐inhibitory *cis* isomer along with MG132 and SiR‐DNA (for blocking anaphase onset and staining mitotic chromosomes, respectively) for 2 h. Then mitotic chromosomes were live imaged (see Section 2). During the live imaging, the inhibitor was switched to the PSS enriched in the inhibitory *trans* isomer by irradiating the cells with 505 nm light. The photo‐switching of the inhibitor either in prometaphase cells that still possessed unaligned chromosomes or before mitotic entry resulted in the formation of misaligned polar chromosomes, demonstrating that the inhibitor was indeed switched to the inhibitory state after the photo‐irradiation (Fig. S6B,C; Video S5). In contrast, photo‐switching of the inhibitor in metaphase cells in which all chromosomes aligned at the equatorial plate, *de novo* misalignment of the pre‐aligned chromosomes was seldom observed in either diploids or tetraploids (Fig. S6D–G; Video S6). This result indicates that aggravation of initially formed misaligned chromosomes rather than failure to maintain pre‐aligned chromosomes is likely to cause extremely prolonged mitosis in CENP‐E‐inhibited tetraploid cells.

### 3.3 Tetraploidy‐linked aggravation of spindle multipolarization and subsequent cell death by paclitaxel treatment

**Fig. 5:**
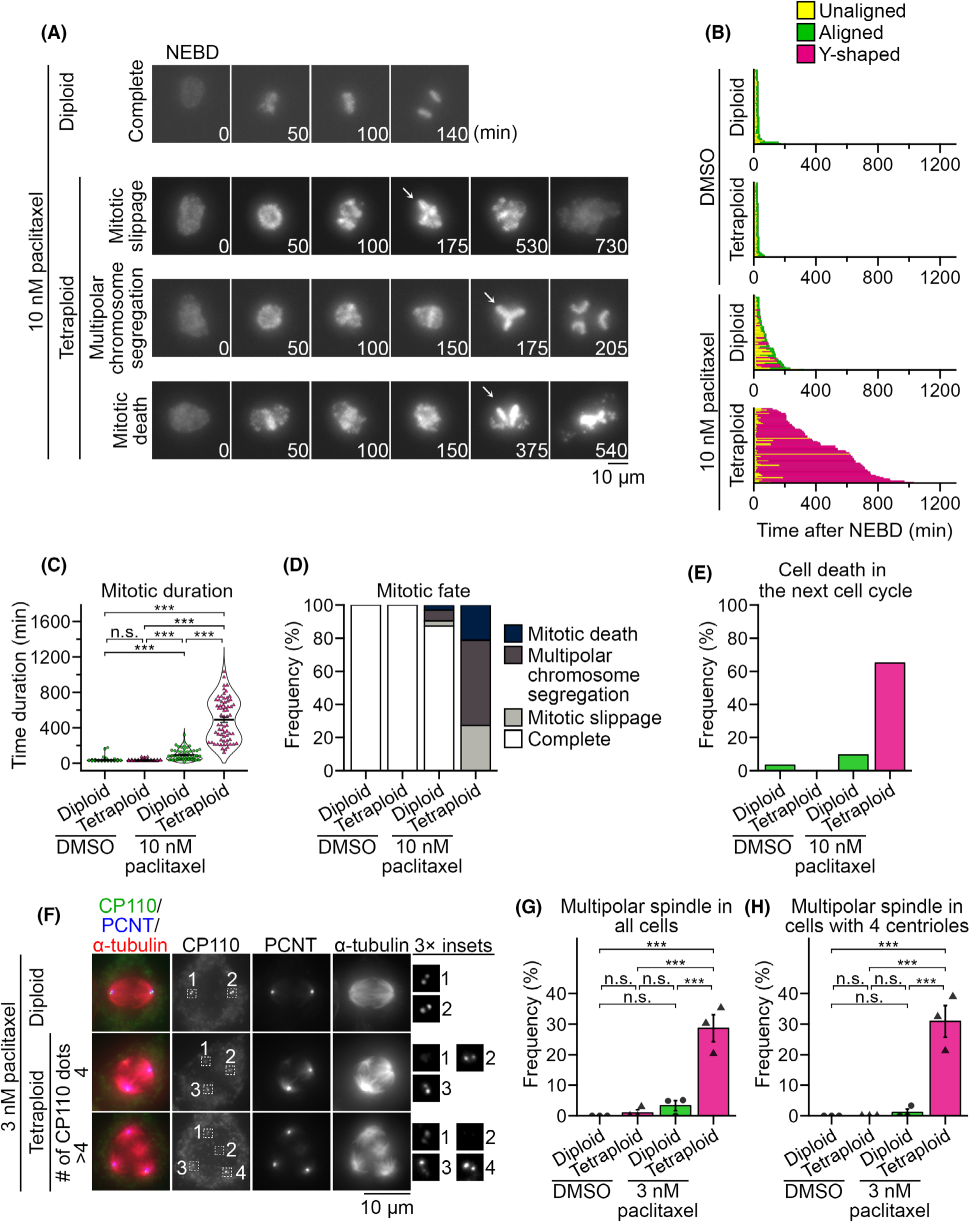
Tetraploidy‐linked aggravation of multipolar spindle formation upon paclitaxel treatment. (A) Time‐lapse images of the mitotic progression in paclitaxel‐treated diploid H2B‐EGFP and tetraploid H2B‐mCherry HAP1 co‐culture. Arrows: Y‐shaped chromosome arrangement. Representative data from two independent experiments. (B) Analysis of mitotic progression of control and paclitaxel‐treated diploid or tetraploid cells in (A). Each bar represents a single mitotic event (from NEBD to anaphase onset or mitotic exit) in a dividing cell. At least 59 cells from two independent experiments were analyzed for each condition. (C) Mitotic duration (from NEBD to anaphase onset or mitotic exit) in control and paclitaxel‐treated diploid or tetraploid cells in (A). Mean ± SE of at least 59 cells from two independent experiments for each condition. Asterisks indicate statistically significant differences between conditions (***P < 0.001, the DSCF test). (D,E) Frequency of mitotic fates (D) or cell death in the subsequent cell cycle (E) in control and paclitaxel‐treated diploid or tetraploid cells in (A). At least 59 and 97 cell, respectively, from two independent experiments were analyzed for each condition in (D) and (E). (F) Immunofluorescence microscopy of CP110, PCNT and α‐tubulin in 3 nm paclitaxel‐treated diploid or tetraploid cells. Representative data from three independent experiments. (G,H) Frequency of multipolar spindle in control and paclitaxel‐treated diploid or tetraploid cells in (F). Data obtained from all cells or only cells with four centrioles are shown in (G) and (H), respectively. Mean ± SE of three independent experiments. At least 92 and 90 cells, respectively, were analyzed for each condition in (G) and (H). Asterisks indicate statistically significant differences between conditions (***P < 0.001, the Steel–Dwass test).

Previous studies revealed that paclitaxel's effects on mitotic control are pleiotropic and concentration‐dependent [34, 35, 36, 37], and cellular processes of the tetraploidy‐selective suppression by paclitaxel remained unclear. To specify paclitaxel‐induced mitotic defects aggravated by tetraploidy and gain insight into the cellular basis of tetraploidy‐selective growth suppression, we compared the effect of paclitaxel on the mitotic progression of co‐cultured diploid and tetraploid cells (Fig. 5A). In the presence of 10 nm paclitaxel, which caused tetraploidy‐selective suppression in co‐culture (Fig. 2C), mitotic progression was significantly delayed in tetraploid cells (with an average mitotic duration of 490 and 32 min in paclitaxel‐ and DMSO‐treated tetraploid cells, respectively; Fig. 5B,C). Paclitaxel‐induced mitotic delay was milder in diploid cells (with an average mitotic duration of 91 and 37 min in paclitaxel‐ and DMSO‐treated diploid cells, respectively; Fig. 5C and Video S7). Importantly, most paclitaxel‐treated tetraploid cells (97%) manifested Y‐shaped abnormal metaphase plates, frequently followed by multipolar chromosome segregation, mitotic death or mitotic slippage (Fig. 5A,B,D; Video S8). The majority of paclitaxel‐treated tetraploid cells that exited mitosis died during the next cell cycle (Fig. 5E). These mitotic defects were much less frequent in paclitaxel‐treated diploids, and most of them underwent normal bipolar chromosome segregation and survived through the next cell cycle (Fig. 5D,E). These results suggest that the tetraploidy‐linked aggravation of multipolar division is a primary cause of tetraploidy‐selective growth suppression by paclitaxel.

Multipolar chromosome segregation accompanying the formation of a ‘Y‐shaped’ metaphase plate suggests spindle multipolarization during pre‐anaphase in the paclitaxel‐treated tetraploid cells. To test this possibility, we conducted immunostaining against α‐tubulin, pericentrin and CP110 (makers of microtubules, pericentriolar material and the centrioles, respectively) in DMSO‐ or 3 nm paclitaxel‐treated diploid or tetraploid cells (Fig. 5F–H). Previously, we found that tetraploid cells suffered chronic centriole overduplication [26]. Therefore, to distinguish the direct influence of tetraploidy on spindle polarity on paclitaxel treatment from an indirect one through the formation of extra centrosomes, we sorted cells based on centriole number per cell in spindle polarity analysis (Fig. 5H). Paclitaxel‐treated tetraploid cells possessed multipolar spindles at a significantly higher frequency than DMSO‐treated control or paclitaxel‐treated diploid cells, both when all cells or only the cells possessing four centrioles were counted (Fig. 5G,H). This result suggests that tetraploidy *per se*, rather than the presence of extra centrosomes, promotes spindle multipolarization upon low concentration paclitaxel treatment, making tetraploid cells more prone to lethal chromosome loss.

### 3.4 CENP‐E inhibitor shows selectivity toward a broader spectrum of tetraploid cell lines than paclitaxel

**Fig. 6:**
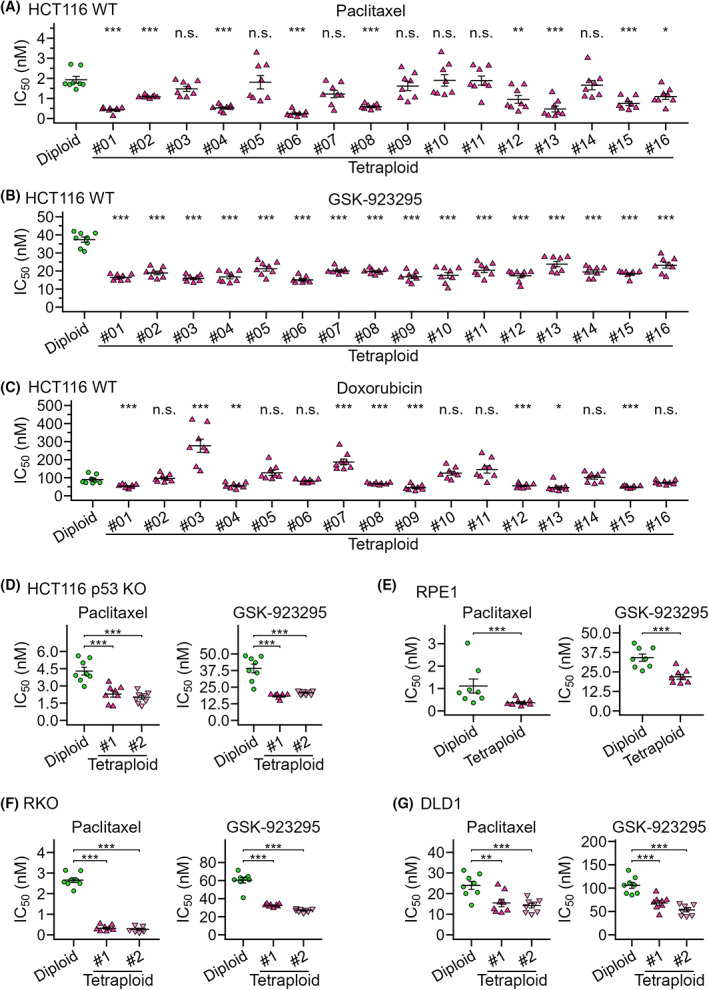
Comparison of efficacy of paclitaxel, GSK‐923295 and doxorubicin between diploids and tetraploids in different cell models. (A–C) IC_50_ values in a comparative colorimetric cell proliferation assay using paclitaxel (A), GSK‐923295 (B) or doxorubicin (C) in diploid or 16 different tetraploid HCT116 cell lines. (D–G) IC_50_ values in a diploid‐tetraploid comparative colorimetric cell proliferation assay in HCT116 p53 knock‐out (D), hTERT‐RPE1 (E), RKO (F) and DLD1 (G) cell models. Mean ± SE of eight replicates from four independent experiments for each condition. Asterisks indicate statistically significant differences in IC_50_ between the control diploid line and each tetraploid line [*P < 0.05, **P < 0.01, ***P < 0.001, the Steel test, except for (E); ***P < 0.001, the Brunner–Munzel test for (E)]. See also Fig. S7 for the dose–response curve of normalized absorbance used to calculate IC_50_.

The above results indicate that CENP‐E inhibitor and paclitaxel selectively suppress tetraploid cell proliferation through different mechanisms, prompting us to compare their effects on tetraploid cells with different cellular backgrounds. For this, we investigated the effect of paclitaxel and GSK‐923295 on the viability of another near‐diploid human cell line, HCT116, and 16 isogenic tetraploid lines (Figs 6A,B, S1B and S7A,B). We also tested the effects of doxorubicin in these cells (Figs 6C and S7C), as a previous study reported tetraploidy‐linked resistance to this drug in the HCT116 background [14]. We found variation in the efficacy of paclitaxel among different tetraploid HCT116 cell lines: although paclitaxel suppressed nine tetraploid cell lines significantly more efficiently than diploid, its IC_50_ values were dispersed among these lines (Fig. 6A). In the remaining seven tetraploid cell lines, the efficacy of paclitaxel did not differ significantly from that in diploids. The response of tetraploid HCT116 lines to doxorubicin was also highly heterogeneous: whereas several tetraploid lines showed a weak trend of or a statistically significant increase in IC_50_ of doxorubicin compared with diploids, others showed significant reductions in IC_50_ (Fig. 6C). These results indicate the limited generality of the ploidy selectivity of paclitaxel or doxorubicin. On the other hand, GSK‐923295 had significantly higher efficacy against all 16 tetraploid HCT116 lines compared with diploids with comparable IC_50_ values (Fig. 6B). We also observed significant tetraploidy selectivity of GSK‐923295 in HCT116 p53 knock‐out background and three additional cell models (hTERT‐RPE1, RKO and DLD1 cells; Figs 6D–G, S1C–F and S7D–G). These data highlight consistent selectivity of CENP‐E inhibition toward tetraploid cells in different backgrounds.

## 4 Discussion

Ploidy alteration causes pleiotropic changes in cell structures and contents, including chromosome number, cell volume and whole‐protein amount, having a profound quantitative effect on mitotic machinery [13, 26, 38]. However, the effects of ploidy alteration on the molecular function of mitotic regulators remain largely unknown. This study reveals that ploidy alteration changes cellular sensitivity to different anti‐mitotic compounds in a complex and non‐uniform manner. Among these compounds, CENP‐E inhibitors showed remarkable and consistent hyperploidy selectivity in mitotic perturbation and cell proliferation suppression through a different mechanism than a previously reported hyperploidy‐selective compound, paclitaxel. CENP‐E inhibition manifested superior consistency to paclitaxel in tetraploidy selectivity across cell lines, suggesting its potential utility in tetraploidy‐specific suppression in a broad spectrum of cellular backgrounds.

Our results indicate that tetraploidy‐linked aggravation of mitotic failure is the leading cause of the sharp tetraploidy selectivity of low‐dose CENP‐E inhibition (Fig. 4). Based on our live imaging, we propose that tetraploidy‐linked aggravation of mitotic failure upon CENP‐E inhibition stems from the combination of (i) tetraploidy‐linked increase in chromosome misalignment and (ii) cohesion fatigue frequently occurring in the time gap between mitotic exit in diploids and tetraploids. To explain point (i) above, we speculate that the doubled chromosome number is the direct cause of the aggravation of chromosome misalignment in CENP‐E‐inhibited tetraploid cells.

A previous study reported that CENP‐E mediates the congression of only a subset of chromosomes located in peripheral areas within the nucleus upon mitotic entry [23]. The doubled chromosome number with the enlarged nucleus in tetraploid cells would increase such peripheral chromosomes vulnerable to CENP‐E inhibition. In unperturbed tetraploid cells, the ‘polar chromosome’ defect was not common (Fig. S5A), indicating that, in normal conditions, CENP‐E sufficiently functions to avoid the prolonged trapping of peripheral chromosomes at the poles, even in tetraploids. However, when CENP‐E was inhibited, a more severe polar chromosome trapping occurred in tetraploids, making them spend significantly longer time to solve chromosome misalignment compared with diploids. This differential effect of CENP‐E inhibition results in a notable time gap between mitotic exit in diploid and tetraploid cells. To explain point (ii) above, cohesion fatigue (premature breakage of sister chromatid cohesion) occurs when mitotic progression is blocked despite continuous tension applied at kinetochores of sister chromatids [39, 40]. Inhibition of CENP‐E motor activity satisfies the criteria for inducing cohesion fatigue with its characteristic effects on mitotic regulations: it blocks the congression of a small proportion of chromatids to block mitotic progression by activating SAC (note that upon inhibition of CENP‐E activity, CENP‐E protein remains at the kinetochores, supporting the recruitment of SAC activation factors) [19, 41], while leaving the majority of chromatids aligned at the metaphase plate under the tension exerted by an intact bipolar spindle [20, 42]. CENP‐E‐inhibited cells typically undergo cohesion fatigue after > 200‐min mitotic arrest (Fig. 4C). By that time, most diploid cells resolve chromosome misalignment and exit mitosis. In contrast, most tetraploid cells remain at mitosis with unsolved chromosome misalignment and undergo irreversible mitotic catastrophe at optimum inhibitor concentration. Based on this model of tetraploidy‐selective suppression, it would be intriguing to address potential ploidy selectivity of different interventions that satisfy the criteria described above: namely, the interventions that differentially modulate mitotic progression among different ploidies while facilitating the occurrence of cohesion fatigue.

We also found that tetraploid cells are more prone to spindle multipolarization than diploid cells are upon paclitaxel treatment. Notably, the paclitaxel concentration most effective for tetraploid‐selective suppression was within the clinically relevant range of the drug concentration [37]. The cause of the tetraploidy‐linked increase in spindle multipolarization remains unknown. Interestingly, a recent study reported that polyploid drosophila embryonic cells were more prone to spindle multipolarization because of the increased steric hindrance of the polyploid amount of chromosomes that precludes supernumerary centrosomes from clustering into bipolar spindle poles [43]. Spindle multipolarization frequently took place even in the tetraploid cells with the normal centrosome number (Fig. 5H), indicating that tetraploidy‐linked aggravation of spindle multipolarity upon paclitaxel treatment occurs by a different mechanism than the one depending on supernumerary centrosomes. We speculate that drastic changes in quantitative features of the mitotic spindle may make tetraploid cells more prone to multipolarize upon paclitaxel treatment. Our results demonstrate that, in contrast to the consistent tetraploidy‐selective trend of CENP‐E inhibitor, the efficacy of paclitaxel and doxorubicin drastically varies even among isogenic HCT116 tetraploid lines. The observed heterogeneous drug response of tetraploid cell lines may at least in part explain the discrepancy in the profiles of ploidy selectivity of paclitaxel or doxorubicin among different studies, including our current study [11, 14, 15]. Future studies on molecular principles that affect the efficacy of these compounds in different tetraploid cell backgrounds would provide further insight into the factors that limit the generalizibility of their ploidy selectivity.

In the HAP1 mixed culture experiments (Fig. 2), we observed the most evident tetraploidy‐selective effects of paclitaxel or GSK‐923295 at concentrations two to three times higher than its IC_50_ obtained in the cell proliferation assays (Fig. 1). A previous study reported a hyperploidy‐selective efficacy of paclitaxel in the haploid‐diploid HAP1 mixed culture at a drug concentration (15 nm) similar to that observed in this study [11] (Fig. 2), and another study reported an IC_50_ value of paclitaxel in HAP1 cells (~ 3 nm) close to the value obtained in this study [44] (Fig. 1). Although the reason for the observed gap between IC_50_ values and the drug concentrations effective for ploidy‐selective suppression is currently unclear, these results indicate that co‐existence of cells with different ploidies has a profound influence on the differential efficacy of these compounds. Future investigation of compound uptake and metabolism among cells with different ploidies in their mixed culture environments may provide an important insight into the principle of the above‐mentioned gap.

## 5 Conclusions

In this study, we found that CENP‐E inhibition is an effective strategy for tetraploidy‐selective cell growth suppression in different cellular backgrounds and culture conditions. Our data showed that CENP‐E inhibition and paclitaxel selectively suppressed tetraploid cell proliferation through different principles from one another and from that of Kif18A‐based tetraploid suppression [12]. These findings imply that quantitative changes in multifaceted aspects of the mitotic regulatory mechanism upon the whole‐genome duplication make tetraploid cells more susceptible to various mitotic perturbations. Moreover, we found that different tetraploidy‐selective interventions cover a different spectrum of tetraploid cellular backgrounds. Taking the high heterogeneity of tetraploid cells into account [5], increasing the choice of drug targets and establishing effective combinations for tetraploid‐selective suppression would benefit cancer therapeutics.

## Conflict of interest

The authors declare no conflict of interest.

## Author contributions

Conceptualization: KYo, MS and RU. Methodology: KYo, AM, MS, KYa, EK, TK, KM, NT, YS, MM and RU. Investigation: KYo, AM, MS, SI, FS, TY and RU. Formal analysis: KYo, AM, MS, SI and RU. Resources: KYo, SI, EK, TK, KM, NT, RS, YS, MM and RU. Writing – Original Draft: KYo and RU. Writing – Review & Editing: KYo, AM, MM and RU. Funding acquisition: KYo, KYa, MM and RU. All authors read and approved the final paper.

### Peer review

The peer review history for this article is available at https://publons.com/publon/10.1002/1878‐0261.13379.

## Supporting information


**Fig. S1.** Flow cytometric DNA content analyses of cell lines used in this study. (A–F) Histograms of Hoechst signal in isogenic ploidy series of HAP1 cells (A), HCT116 cells (B), HCT116 p53 knock‐out cells (C), hTERT‐RPE1 cells (D), RKO cells (E) or DLD1 cells (F). Representative data from two independent experiments. The absence of p53 protein in HCT116 p53 knock‐out cells was confirmed by immunoblotting (C; representative data from three independent experiments).

**Fig. S2.** Efficacy of anti‐mitotic compounds in haploid, diploid and tetraploid HAP1 cells. (A) Dose–response curve of normalized absorbance in a comparative colorimetric cell proliferation assay using different anti‐mitotic compounds in haploid, diploid and tetraploid HAP1 cells. Unit of inhibitor concentration is shown at the top of each graph. (B) IC_50_ values of anti‐mitotic compounds in haploid, diploid and tetraploid HAP1 cells (symbolized as H, D and T, respectively), calculated from the dose–response curves in (A). Mean ± SE of eight replicates from four independent experiments for each condition. Asterisks indicate statistically significant differences in IC_50_ between cells with different ploidies (*P < 0.05, **P < 0.01, ***P < 0.001, the Steel–Dwass test). The identical data on paclitaxel, GSK‐923295, STLC and doxorubicin are also shown in Fig. 1A.

**Fig. S3.** Selective anti‐proliferative effect of paclitaxel and CENP‐E inhibitors on two independent HAP1 tetraploid cell lines. (A,B) Dose–response curve of normalized absorbance (A) and calculated drug IC_50_ values (B) in a comparative colorimetric cell proliferation assay using paclitaxel, CENP‐E inhibitors or doxorubicin in diploid and two different tetraploid HAP1 cell lines. Mean ± SE of eight replicates from four independent experiments for each condition. Asterisks indicate statistically significant differences in IC_50_ between cells with different ploidies (***P < 0.001, the Steel test).

**Fig. S4.** Tetraploidy‐selective effects of paclitaxel or GSK‐923295 in diploid‐tetraploid HAP1 co‐culture. (A,C) Flow cytometric analyses of diploid and tetraploid cell numbers in their co‐culture treated with paclitaxel for 48 h (A) or paclitaxel or GSK‐923295 for the longer term (C). Dot plots of EGFP intensity against the Hoechst signal or histograms of the Hoechst signal are shown at top and bottom, respectively. Cell populations originating from diploid or tetraploid cells were distinguished based on EGFP signal intensity and separately displayed in the histograms. (B) The proportion of tetraploid cells in the diploid‐tetraploid co‐culture. Mean ± SE of three independent experiments for each condition. Asterisks indicate statistically significant differences between conditions (***P < 0.001, the Steel–Dwass test).

**Fig. S5.** Gradual re‐alignment of misaligned polar chromosomes in GSK‐923295‐treated cells. (A) Left: Immunostaining microscopy of α‐tubulin in mitotic tetraploid HAP1 cells in unperturbed asynchronous culture. DNA was stained with DAPI. Right: Frequency of pre‐anaphase mitotic stage or polar chromosome phenotype. Mean ± SE of three independent experiments. At least 222 mitotic cells were analyzed for each condition. There was no statistically significant difference between diploids and tetraploids (the Brunner–Munzel test). (B,C) GSK‐923295‐treated tetraploid cells whose polar chromosomes gradually moved into the metaphase plate (B; type 1) or did not undergo re‐alignment (C; type 2). Arrowheads: misaligned polar chromosomes. Arrows: Gross chromosome scattering caused through cohesion fatigue. (D) Frequency of different types of misaligned chromosome movement before cohesion fatigue in GSK‐923295‐treated diploid or tetraploid cells. Cells that underwent cohesion fatigue were analyzed from the results of two independent experiments.

**Fig. S6.** CENP‐E inhibition does not impair the maintenance of the pre‐aligned metaphase chromosomes. (A) Photoisomerization of the photo‐switchable CENP‐E inhibitor, PCEI‐HU. (B,D,F) Schemes (top) and time‐lapse images (bottom) of mitotic progression in HAP1 cells treated with DMSO or PCEI‐HU. Cells were pre‐treated with MG132 and SiR‐DNA for blocking anaphase onset and staining chromosomes, respectively. Photo‐switching of the inhibitor from the non‐inhibitory PSS_365_ to inhibitory PSS_505_ was induced before or after the completion of chromosome alignment in B or D, respectively. Note that the inhibitor blocked the equatorward movement of the misaligned polar chromosomes at PSS_505_ (B), whereas it did not affect the maintenance of the pre‐aligned chromosomes (D). Cells that entered mitosis within 85 min after photo‐irradiation (and, therefore, contained misaligned chromosomes in the presence of PCEI‐HU at PSS_505_) were included in the category shown in (B). For comparison, we also tested chromosome movement in the cells treated with the inhibitor at PSS_365_ throughout the live imaging (F). * Neighboring cells. (C) Frequency of interphase cells, or mitotic cells with misaligned or aligned chromosomes in (B). At least 13 cells pooled from three independent experiments were analyzed for each condition. (E,G) Cumulative frequency of *de novo* misalignment of the pre‐aligned chromosomes in (D) or (F) (E or G, respectively). Mean ± SE of at least 44 cells from three independent experiments (n.s. between diploid and tetraploid cells at 110 min, the Brunner–Munzel test). Note that *de novo* misalignment was infrequent in diploids and tetraploids in all conditions.

**Fig. S7.** Proliferation of diploids or tetraploids in different cell models treated with different concentrations of paclitaxel, GSK‐923295 or doxorubicin. (A–C) Dose–response curve of normalized absorbance in a comparative colorimetric cell proliferation assay using paclitaxel (A), GSK‐923295 (B) or doxorubicin (C) in diploid and tetraploid HCT116 cells. (D–G) Dose–response curve of normalized absorbance in a diploid‐tetraploid comparative colorimetric cell proliferation assay in HCT116 p53 knock‐out (D), hTERT‐RPE1 (E), RKO (F) or DLD1 (G) cell models. Mean ± SE of eight replicates from four independent experiments for each condition. To facilitate the comparison, identical dose–response plots of diploids were overlaid in all graphs of tetraploid plots in (A–C).

**Table S1.** Supporting data for cell proliferation assays. All datasets were obtained in cell proliferation assays and all coefficients and constant values were obtained by the curve fitting.

**Video S1.** Mitotic progression of DMSO‐treated diploid HAP1 cells. Maximum projected fluorescence microscopy of diploid HAP1 H2B‐EGFP cells treated with DMSO. The movie is shown at 1500× real‐time. Scale bar: 10 μm.

**Video S2.** Mitotic progression of DMSO‐treated tetraploid HAP1 cells. Maximum projected fluorescence microscopy of tetraploid HAP1 H2B‐mCherry cells treated with DMSO. The movie is shown at 1500× real‐time. Scale bar: 10 μm.

**Video S3.** Mitotic progression in GSK‐923295‐treated diploid HAP1 cells. Maximum projected fluorescence microscopy of diploid HAP1 H2B‐EGFP cells treated with GSK‐923295. The movie is shown at 1500× real‐time. Scale bar: 10 μm.

**Video S4.** Mitotic progression in GSK‐923295‐treated tetraploid HAP1 cells. Maximum projected fluorescence microscopy of tetraploid HAP1 H2B‐mCherry cells treated with GSK‐923295. The movie is shown at 1500× real‐time. Scale bar: 10 μm.

**Video S5.** Mitotic progression in PCEI‐HU‐treated tetraploid HAP1 cells. Fluorescence microscopy of SiR‐DNA‐stained cells. The movie is shown at 900× real‐time. Scale bar: 10 μm. PCEI‐HU was at PSS_365 nm_ (magenta boxes) or PSS_505 nm_ (cyan boxes) in the time frames. Note that the sample was irradiated with 505 nm light (between time frames #3 and 4) before cells completed chromosome alignment.

**Video S6.** Mitotic progression in PCEI‐HU‐treated tetraploid HAP1 cells. Fluorescence microscopy of SiR‐DNA‐stained cells. The movie is shown at 900× real‐time. Scale bar: 10 μm. PCEI‐HU was at PSS_365 nm_ (magenta boxes) or PSS_505 nm_ (cyan boxes) in the time frames. Note that the sample was irradiated with 505 nm light (between time frames #3 and 4) after cells completed chromosome alignment.

**Video S7.** Mitotic progression in paclitaxel‐treated diploid HAP1 cells. Maximum projected fluorescence microscopy of diploid HAP1 H2B‐EGFP cells treated with paclitaxel. The movie is shown at 1500 × real‐time. Scale bar: 10 μm.

**Video S8.** Mitotic progression in paclitaxel‐treated tetraploid HAP1 cells. Maximum projected fluorescence microscopy of tetraploid HAP1 H2B‐mCherry cells treated with paclitaxel. The movie is shown at 1500× real‐time. Scale bar: 10 μm.

## Data Availability

The data supporting the findings of this study are provided in the Supplementary Information of this article or are available upon reasonable request.
