# Supplementary figures and images for: Tetraploidy‐linked sensitization to CENP‐E inhibition in human cells
Source: Mol Oncol. 2023 Feb 11;17(6):1148–66. doi: 10.1002/1878-0261.13379 (PMC10257419; doi:10.1002/1878-0261.13379)

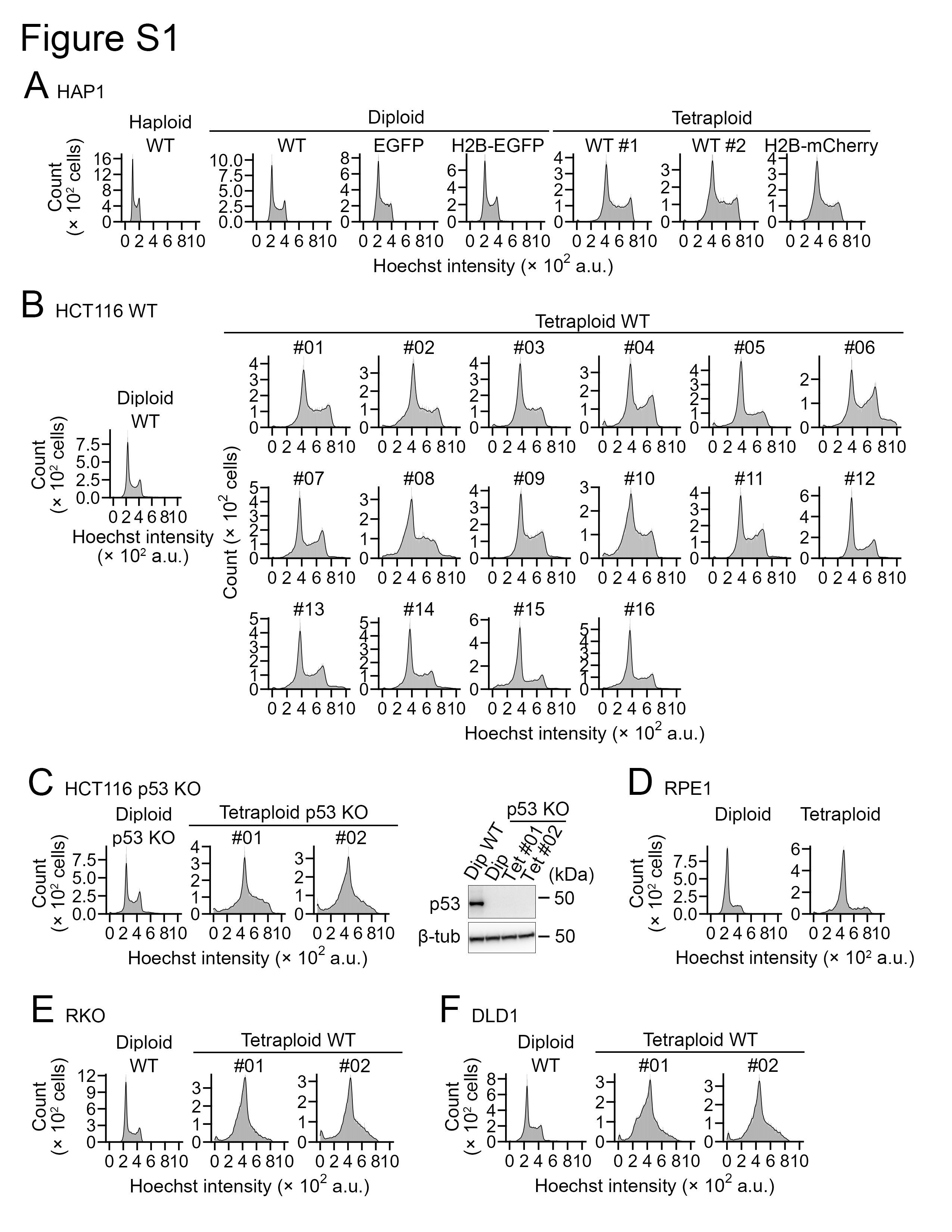

Supplement: Supplementary file 1 — Fig. S1. Flow cytometric DNA content analyses of cell lines used in this study. (A–F) Histograms of Hoechst signal in isogenic ploidy series of HAP1 cells (A), HCT116 cells (B), HCT116 p53 knock‐out cells (C), hTERT‐RPE1 cells (D), RKO cells (E) or DLD1 cells (F). Representative data from two independent experiments. The absence of p53 protein in HCT116 p53 knock‐out cells was confirmed by immunoblotting (C; representative data from three independent experiments). [file MOL2-17-1148-s008.tif]

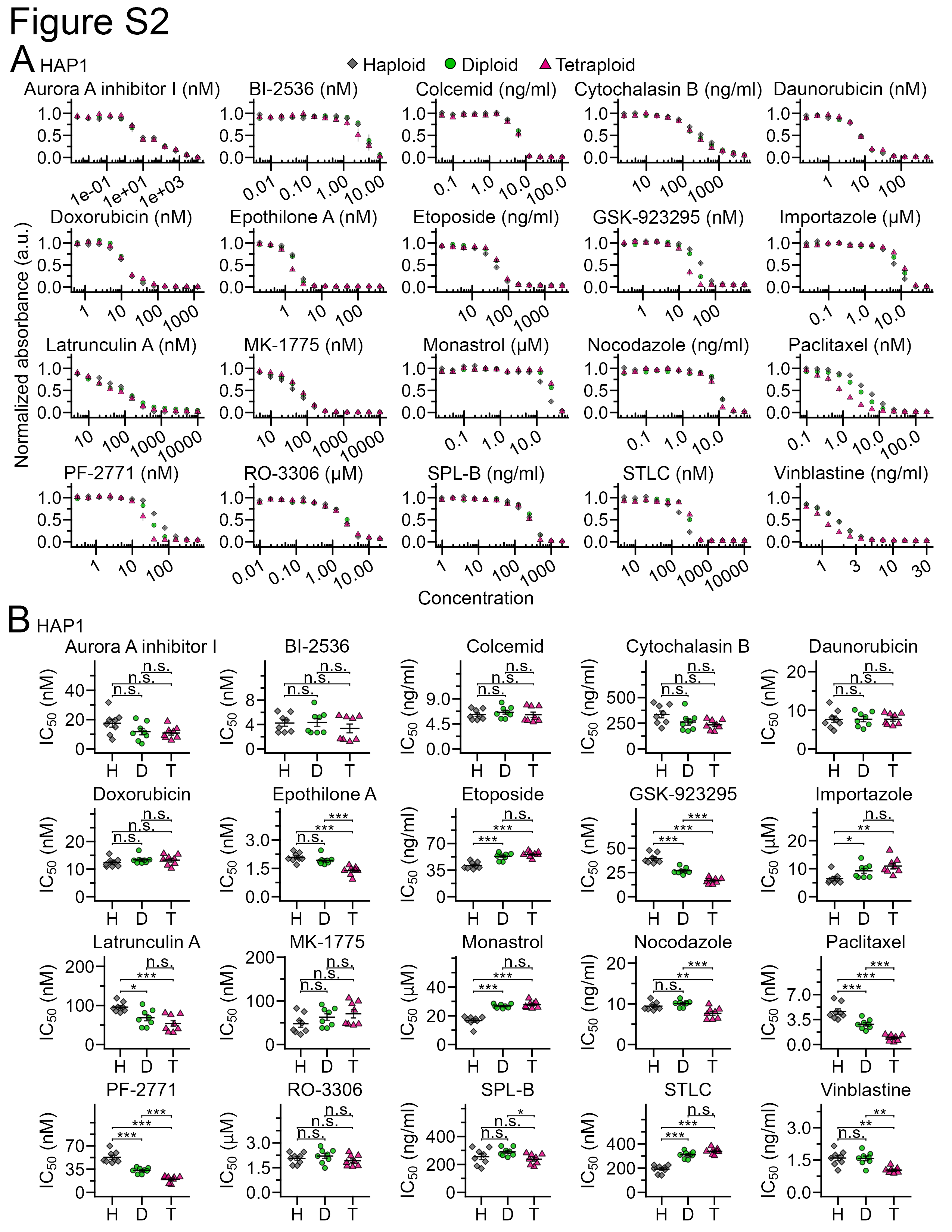

Supplement: Supplementary file 2 — Fig. S2. Efficacy of anti‐mitotic compounds in haploid, diploid and tetraploid HAP1 cells. (A) Dose–response curve of normalized absorbance in a comparative colorimetric cell proliferation assay using different anti‐mitotic compounds in haploid, diploid and tetraploid HAP1 cells. Unit of inhibitor concentration is shown at the top of each graph. (B) IC50 values of anti‐mitotic compounds in haploid, diploid and tetraploid HAP1 cells (symbolized as H, D and T, respectively), calculated from the dose–response curves in (A). Mean ± SE of eight replicates from four independent experiments for each condition. Asterisks indicate statistically significant differences in IC50 between cells with different ploidies (*P < 0.05, **P < 0.01, ***P < 0.001, the Steel–Dwass test). The identical data on paclitaxel, GSK‐923295, STLC and doxorubicin are also shown in Fig. 1A. [file MOL2-17-1148-s010.tif]

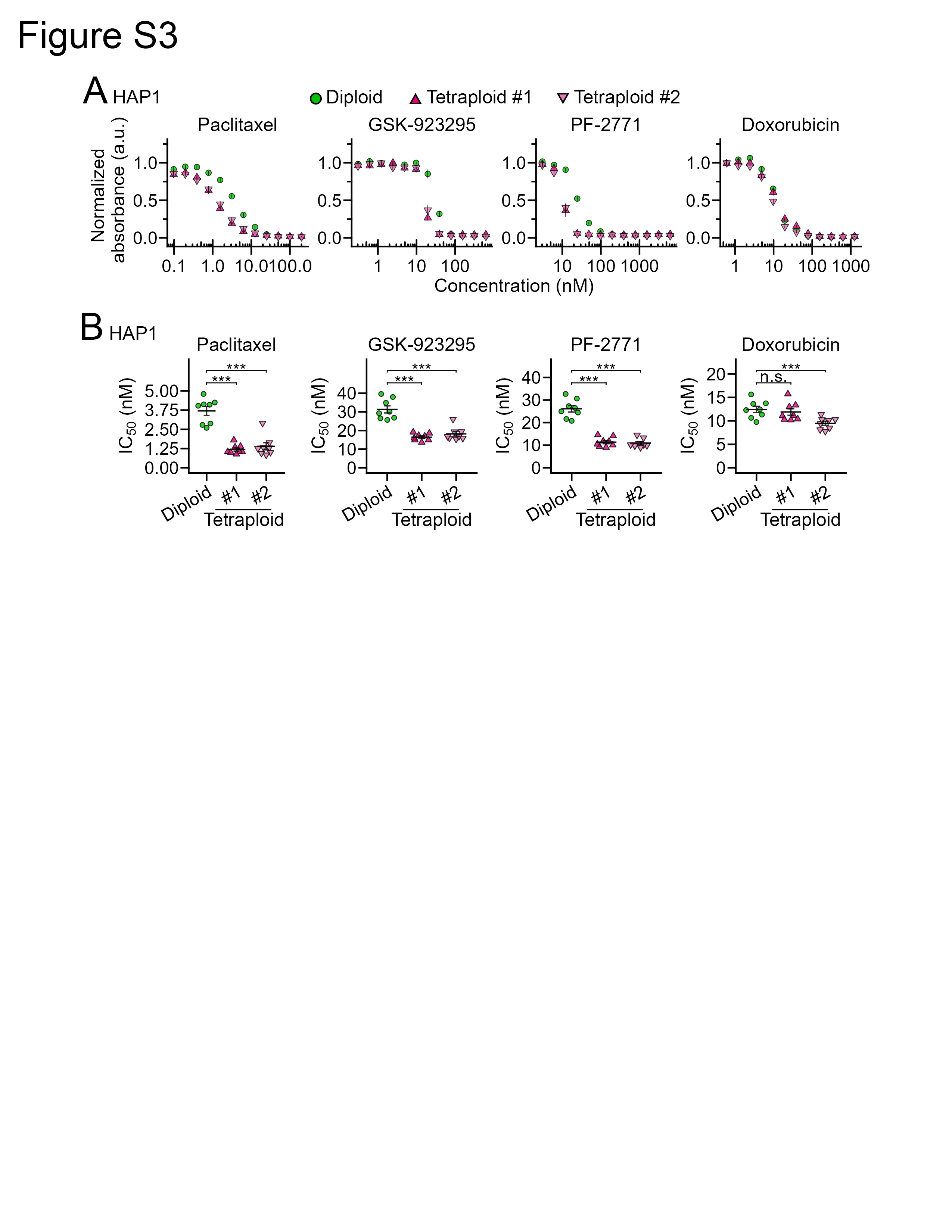

Supplement: Supplementary file 3 — Fig. S3. Selective anti‐proliferative effect of paclitaxel and CENP‐E inhibitors on two independent HAP1 tetraploid cell lines. (A,B) Dose–response curve of normalized absorbance (A) and calculated drug IC50 values (B) in a comparative colorimetric cell proliferation assay using paclitaxel, CENP‐E inhibitors or doxorubicin in diploid and two different tetraploid HAP1 cell lines. Mean ± SE of eight replicates from four independent experiments for each condition. Asterisks indicate statistically significant differences in IC50 between cells with different ploidies (***P < 0.001, the Steel test). [file MOL2-17-1148-s013.tif]

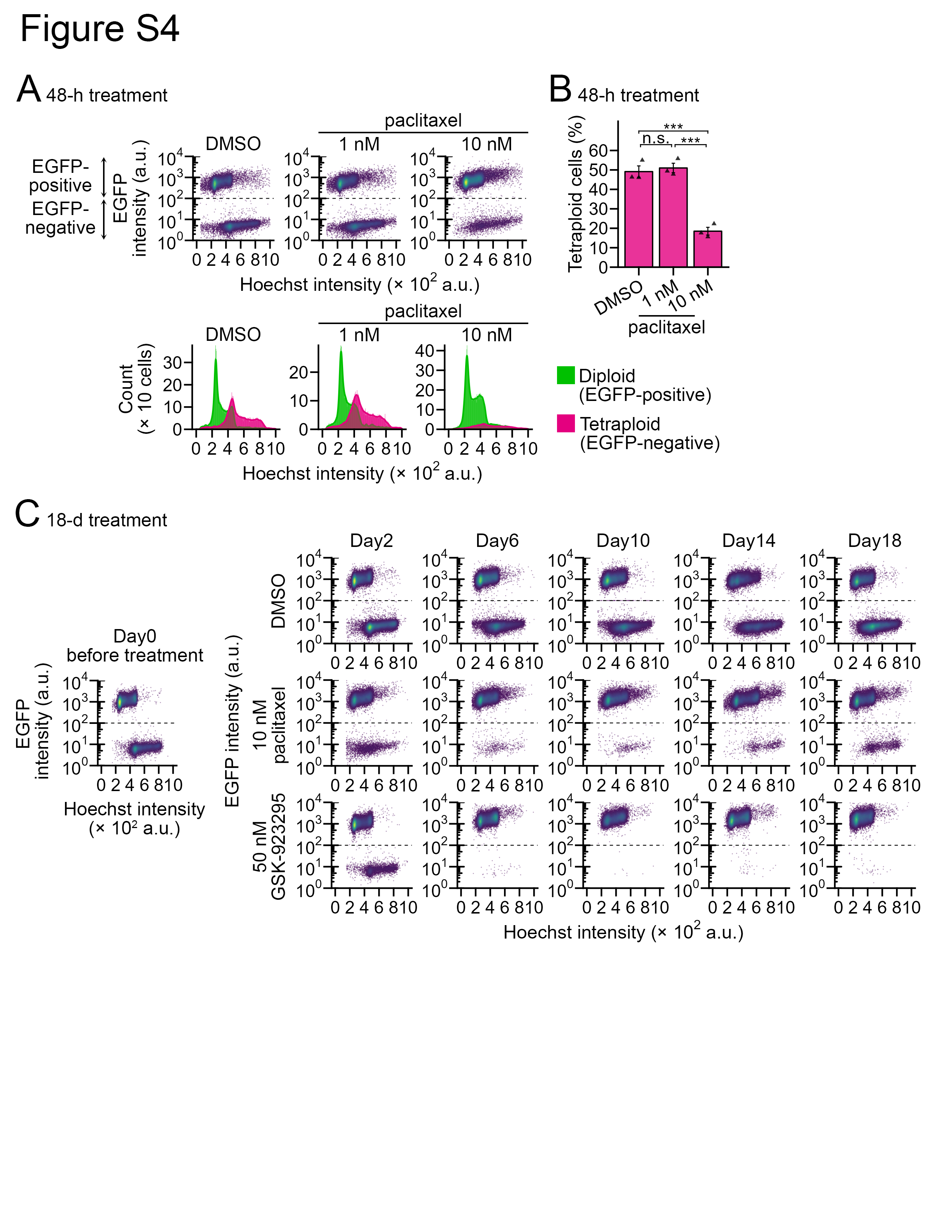

Supplement: Supplementary file 4 — Fig. S4. Tetraploidy‐selective effects of paclitaxel or GSK‐923295 in diploid‐tetraploid HAP1 co‐culture. (A,C) Flow cytometric analyses of diploid and tetraploid cell numbers in their co‐culture treated with paclitaxel for 48 h (A) or paclitaxel or GSK‐923295 for the longer term (C). Dot plots of EGFP intensity against the Hoechst signal or histograms of the Hoechst signal are shown at top and bottom, respectively. Cell populations originating from diploid or tetraploid cells were distinguished based on EGFP signal intensity and separately displayed in the histograms. (B) The proportion of tetraploid cells in the diploid‐tetraploid co‐culture. Mean ± SE of three independent experiments for each condition. Asterisks indicate statistically significant differences between conditions (***P < 0.001, the Steel–Dwass test). [file MOL2-17-1148-s015.tif]

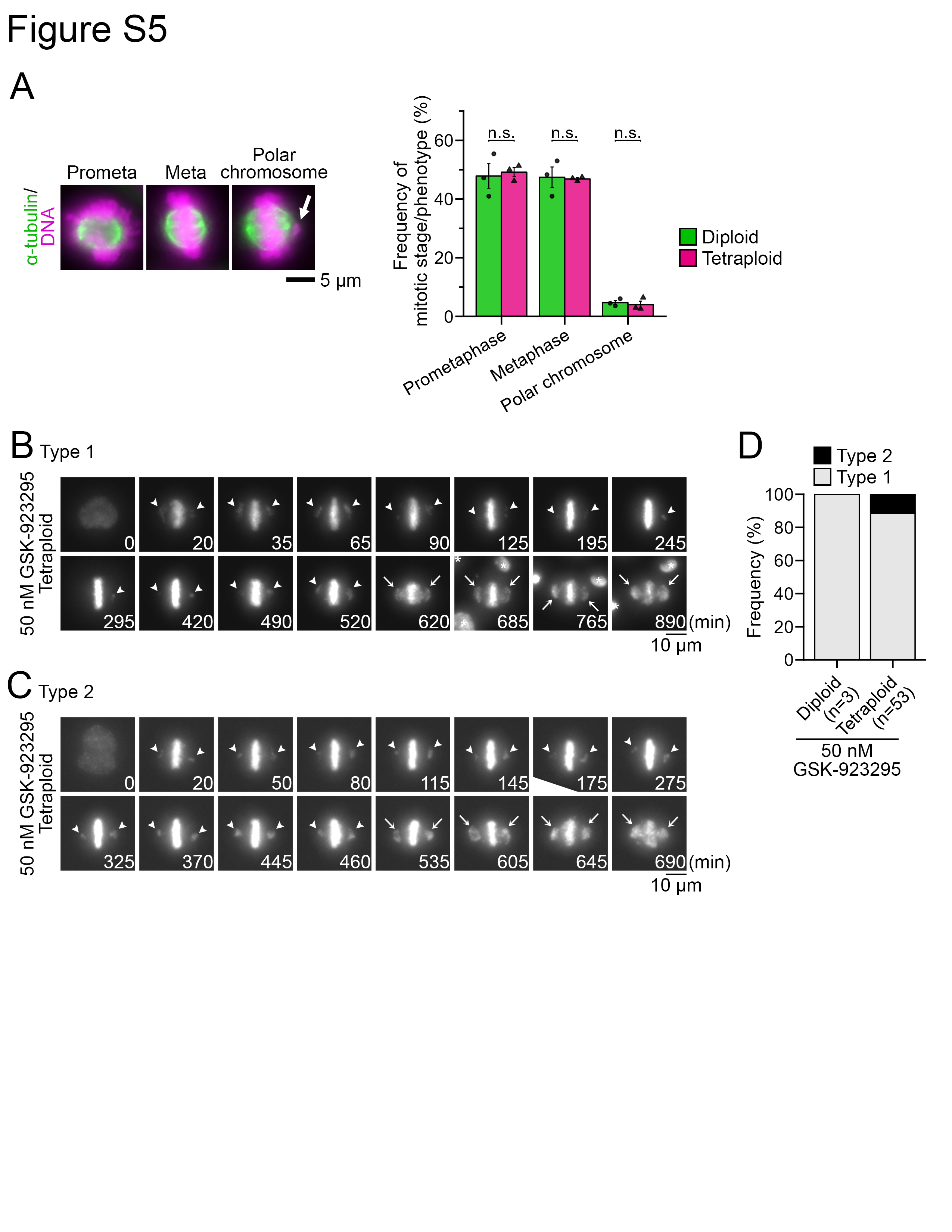

Supplement: Supplementary file 5 — Fig. S5. Gradual re‐alignment of misaligned polar chromosomes in GSK‐923295‐treated cells. (A) Left: Immunostaining microscopy of α‐tubulin in mitotic tetraploid HAP1 cells in unperturbed asynchronous culture. DNA was stained with DAPI. Right: Frequency of pre‐anaphase mitotic stage or polar chromosome phenotype. Mean ± SE of three independent experiments. At least 222 mitotic cells were analyzed for each condition. There was no statistically significant difference between diploids and tetraploids (the Brunner–Munzel test). (B,C) GSK‐923295‐treated tetraploid cells whose polar chromosomes gradually moved into the metaphase plate (B; type 1) or did not undergo re‐alignment (C; type 2). Arrowheads: misaligned polar chromosomes. Arrows: Gross chromosome scattering caused through cohesion fatigue. (D) Frequency of different types of misaligned chromosome movement before cohesion fatigue in GSK‐923295‐treated diploid or tetraploid cells. Cells that underwent cohesion fatigue were analyzed from the results of two independent experiments. [file MOL2-17-1148-s001.tif]

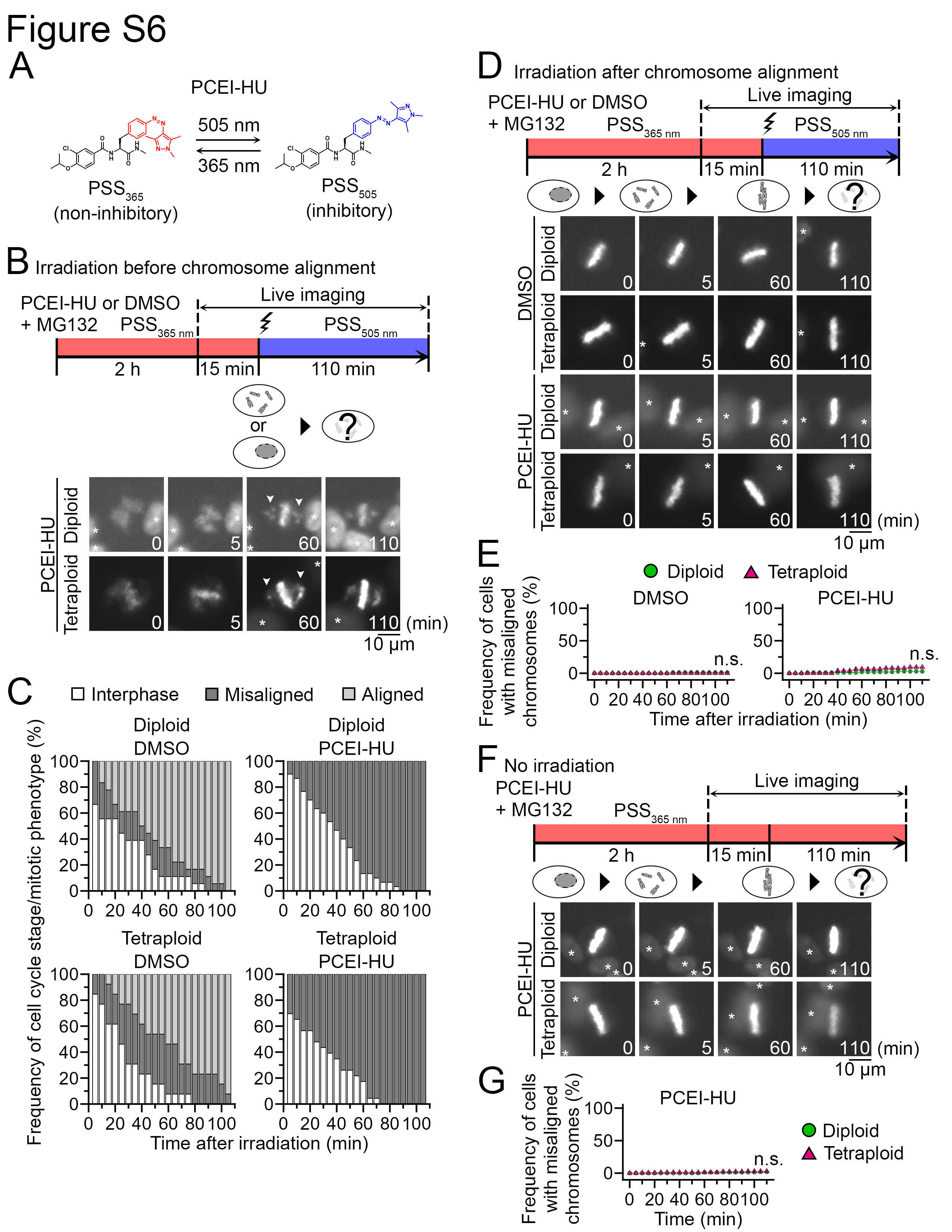

Supplement: Supplementary file 6 — Fig. S6. CENP‐E inhibition does not impair the maintenance of the pre‐aligned metaphase chromosomes. (A) Photoisomerization of the photo‐switchable CENP‐E inhibitor, PCEI‐HU. (B,D,F) Schemes (top) and time‐lapse images (bottom) of mitotic progression in HAP1 cells treated with DMSO or PCEI‐HU. Cells were pre‐treated with MG132 and SiR‐DNA for blocking anaphase onset and staining chromosomes, respectively. Photo‐switching of the inhibitor from the non‐inhibitory PSS365 to inhibitory PSS505 was induced before or after the completion of chromosome alignment in B or D, respectively. Note that the inhibitor blocked the equatorward movement of the misaligned polar chromosomes at PSS505 (B), whereas it did not affect the maintenance of the pre‐aligned chromosomes (D). Cells that entered mitosis within 85 min after photo‐irradiation (and, therefore, contained misaligned chromosomes in the presence of PCEI‐HU at PSS505) were included in the category shown in (B). For comparison, we also tested chromosome movement in the cells treated with the inhibitor at PSS365 throughout the live imaging (F). * Neighboring cells. (C) Frequency of interphase cells, or mitotic cells with misaligned or aligned chromosomes in (B). At least 13 cells pooled from three independent experiments were analyzed for each condition. (E,G) Cumulative frequency of de novo misalignment of the pre‐aligned chromosomes in (D) or (F) (E or G, respectively). Mean ± SE of at least 44 cells from three independent experiments (n.s. between diploid and tetraploid cells at 110 min, the Brunner–Munzel test). Note that de novo misalignment was infrequent in diploids and tetraploids in all conditions. [file MOL2-17-1148-s009.tif]

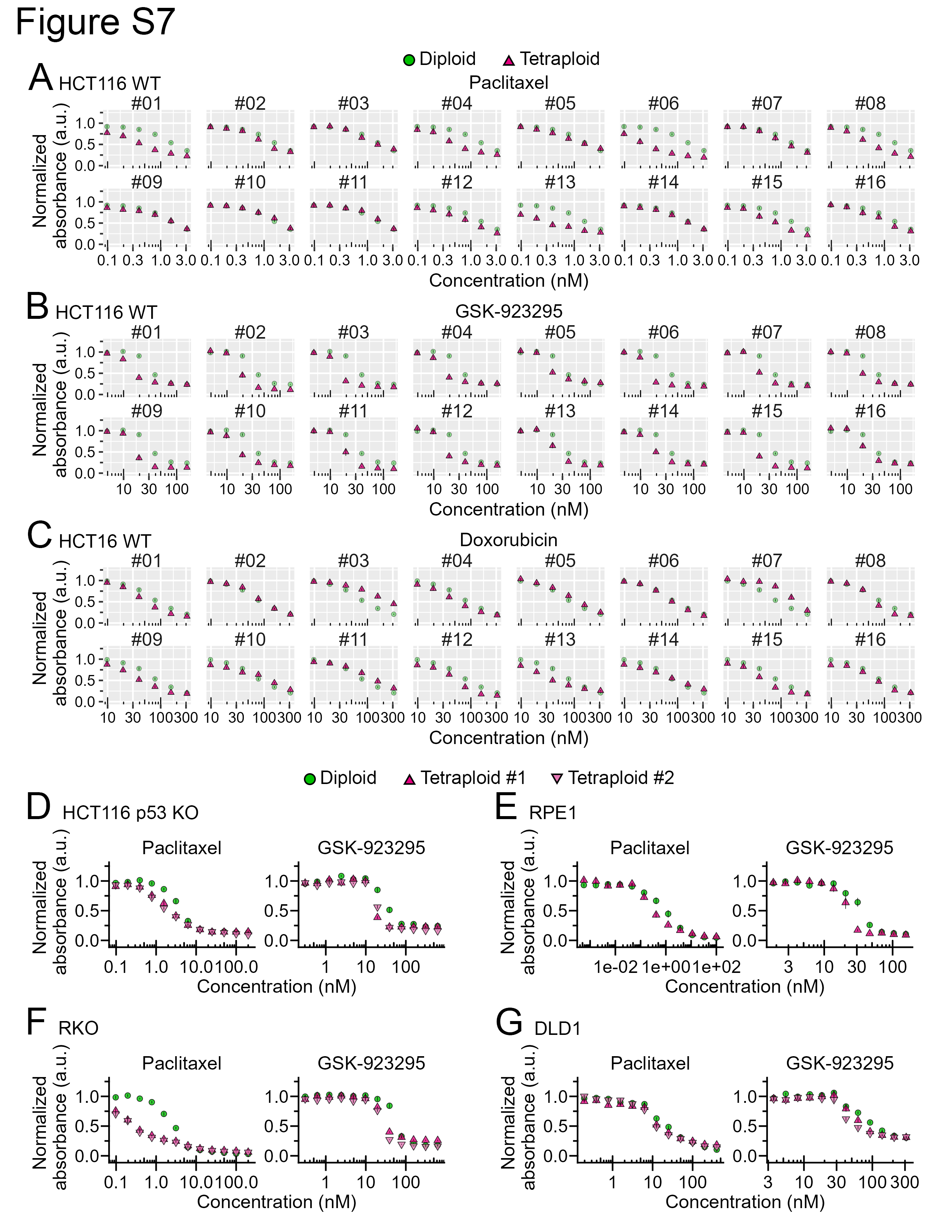

Supplement: Supplementary file 7 — Fig. S7. Proliferation of diploids or tetraploids in different cell models treated with different concentrations of paclitaxel, GSK‐923295 or doxorubicin. (A–C) Dose–response curve of normalized absorbance in a comparative colorimetric cell proliferation assay using paclitaxel (A), GSK‐923295 (B) or doxorubicin (C) in diploid and tetraploid HCT116 cells. (D–G) Dose–response curve of normalized absorbance in a diploid‐tetraploid comparative colorimetric cell proliferation assay in HCT116 p53 knock‐out (D), hTERT‐RPE1 (E), RKO (F) or DLD1 (G) cell models. Mean ± SE of eight replicates from four independent experiments for each condition. To facilitate the comparison, identical dose–response plots of diploids were overlaid in all graphs of tetraploid plots in (A–C). [file MOL2-17-1148-s005.tif]
