# Supplementary material for: Tetraploidy‐linked sensitization to CENP‐E inhibition in human cells
Source: Mol Oncol. 2023 Feb 11;17(6):1148–66. doi: 10.1002/1878-0261.13379 (PMC10257419; doi:10.1002/1878-0261.13379)
Supplement: Supplementary file 8 — Table S1. Supporting data for cell proliferation assays. All datasets were obtained in cell proliferation assays and all coefficients and constant values were obtained by the curve fitting. [file MOL2-17-1148-s006.docx]

**Supplementary Table 1: Supporting data for cell proliferation assays**

All datasets obtained in cell proliferation assays and all coefficients and constant values obtained by the curve fitting.
